# Facile and Stereo-Selective Synthesis of UDP-α-D-xylose and UDP-β-L-arabinose Using UDP-Sugar Pyrophosphorylase

**DOI:** 10.3389/fchem.2018.00163

**Published:** 2018-05-23

**Authors:** JiaJia Wang, Harmon Greenway, Shanshan Li, Mohui Wei, Samuel J. Polizzi, Peng G. Wang

**Affiliations:** ^1^Joint National Laboratory for Antibody Drug Engineering, School of Basic Medical Science, Henan University, Kaifeng, China; ^2^Department of Chemistry and Center for Diagnostics and Therapeutics, Georgia State University, Atlanta, GA, United States; ^3^Chemily, LLC, Atlanta, GA, United States

**Keywords:** UDP-xylose, UDP-arabinose, nucleotide sugar, chemo-enzymatic synthesis, UDP-sugar pyrophosphorylase

## Abstract

A novel synthesis of nucleotide sugars was conducted to prepare UDP-α-D-xylose and UDP-β-L-arabinose without utilizing protection strategies or advanced purification techniques. Sugar-1-phosphates of D-xylose and L-arabinose were synthesized from their β-glycosylsulfonylhydrazides and evaluated as substrates for recombinant UDP-sugar pyrophosphorylases from *Arabidopsis thaliana* or *Bifidobacterium infantis* to furnish the biologically active nucleotide. The facile, three-step procedure takes advantage of substrate diversity available through chemical synthesis followed by the selectivity of enzyme catalysis. This approach increases the substrate scope of enzymatic preparation and expands access to stereopure nucleotide sugars on preparative scale. Increased production of both sugars has implications for glycoengineering and glycan production using glycosyltransferases.

## Introduction

The nucleotide sugars UDP-α-D-xylose (UDP-Xyl) and UDP-β-L-arabinose (UDP-Ara) are required substrates in the corresponding glycosyltransferase (GT)-catalyzed synthesis of polysaccharides comprising important biopolymers in animal and plant species. For example, UDP-Xyl is essential for the xylosylation of Notch signaling receptors (Lee et al., 2013), and initiates the core protein linkage in the proteoglycans heparin, chondroitin and dermatan sulfate (Esko et al., 2009; Beahm et al., 2014). UDP-Ara is a donor molecule for structural extensin glycoproteins in plant cell walls (Chen et al., 2015), and arabinose chains on therapeutic antibodies expressed in plant-based systems (Dicker et al., 2016). Despite their role in diverse and health-related processes, convenient preparations of UDP-Xyl and UDP-Ara are still not generally well-established and ready to meet the needs of a growing glycoscience community (Pauly et al., 2000; Kotake et al., 2009; Damerow et al., 2010; Yang and Bar-Peled, 2010; Gu et al., 2011).

In our own studies of glycan maturation and glycoengineering, we have developed a need for diverse nucleotide sugars. Nucleotide sugars have been prepared by chemical synthetic methods, including those based on Khorana's use of phosphoromorpholidates for pyrophosphate formation (Roseman et al., 1961), and Chi–Huey Wong's work with dibenzyl N,N-diethylphosphoramidite as a phosphitylating agent (Sim et al., 1993). Specific to the current study, UDP-Xyl has previously been prepared using a phospho-imidazolidate activation method (Ishimizu et al., 2005), and both UDP-Xyl and UDP-Ara have been produced using 1,2-anhydro sugars (Ernst and Klaffke, 2003). However, chemical methods for the preparation of nucleotide sugars are widely reviewed as difficult to reproduce, multi-step procedures with advanced purification requirements and low to moderate isolated yields (Wagner et al., 2009). Additionally, chemical synthesis of nucleotide sugars often requires protection strategies in order to selectively obtain the biologically useful anomer of the activated sugar (Zhang and Liu, 2000; Ernst and Klaffke, 2003; Wolf et al., 2011).

Advances in the enzymatic synthesis of nucleotide sugars by our group and others have expanded access to a wide range of nucleoside-diphosphate (NDP) sugars (Guan et al., 2009; Li et al., 2015; Muthana et al., 2015; Yu and Chen, 2016). These preparations utilize enzymes from sugar recycling, salvage pathways, typically a sugar-1-phosphate kinase and a promiscuous NDP-sugar pyrophosphorylase, to give high yields of anomerically pure nucleotide sugars in a one-pot multi-enzyme (OPME) system (Muthana et al., 2012; Li et al., 2013). However, these preparations are generally limited by the substrate specificity of available enzyme combinations, and particularly by kinases reported to be more substrate specific than the promiscuous NDP pyrophosphorylases (Errey et al., 2004).

**Figure 1:**
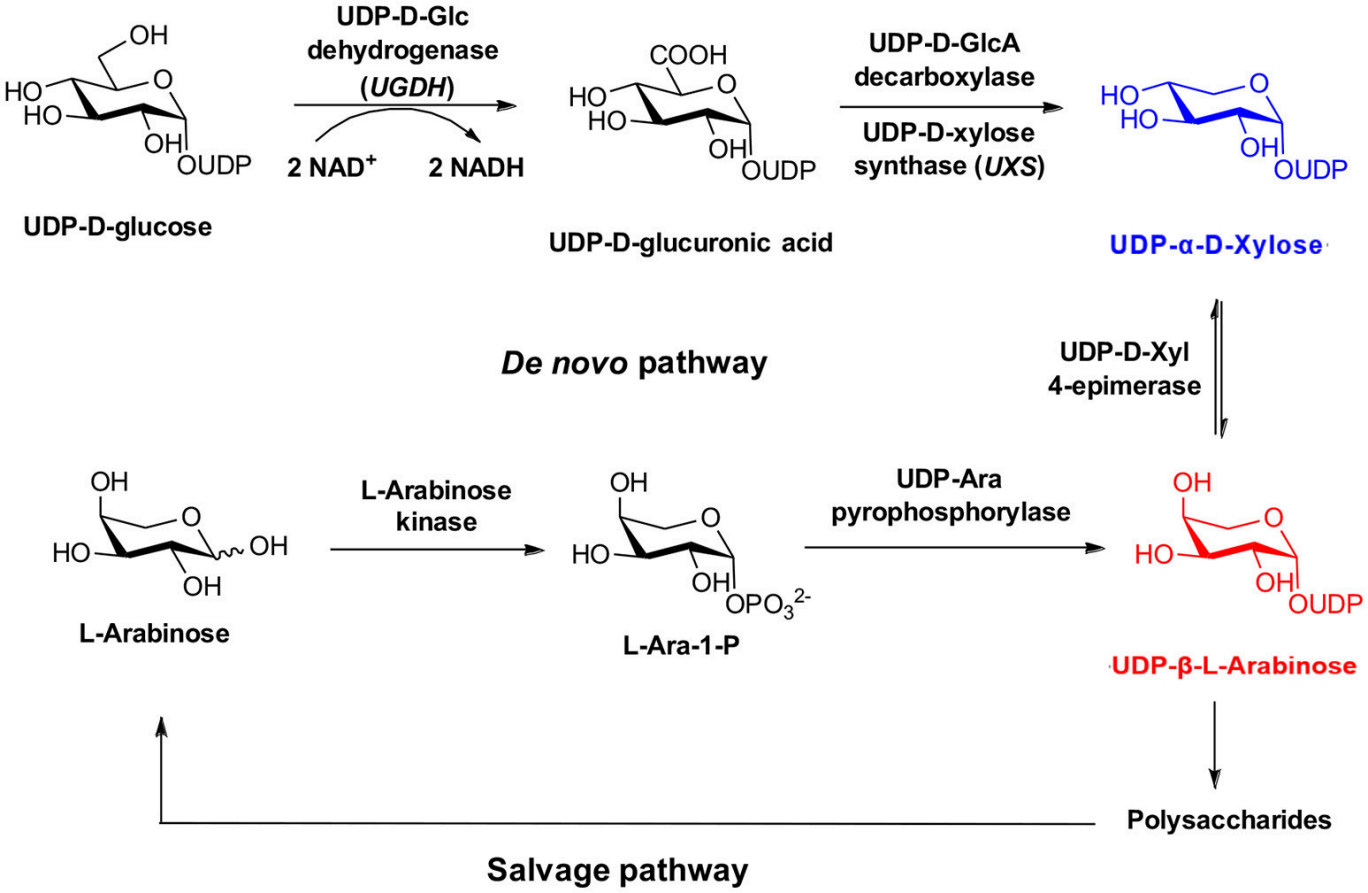
Biosynthesis of UDP-α-D-Xylose and UDP-β-L-Arabinose.

In the case of UDP-Xyl, a salvage pathway for recycling of D-xylose into stereopure nucleotide sugars has not been established, and the existence of a kinase responsible for biosynthesis of an intermediate xylose-1-phosphate is currently hypothetical (Geserick and Tenhaken, 2013). For this reason attention has focused on the *de novo* biosynthetic pathway (Figure 1), starting with the two-step oxidation of UDP-Glc to UDP-GlcA by UDP-glucose dehydrogenase (UGDH), and subsequent oxidative decarboxylation by UDP-Xyl synthase (UXS, a.k.a. UDP-GlcA decarboxylase) (Harper and Bar-Peled, 2002). However, unlike the salvage pathway, a OPME strategy using UGDH/UXS requires exogenous NAD^+^ cofactor, and is complicated by diverse inhibition mechanisms, including hysteresis, and competitive and allosteric inhibition of UGDH (Kadirvelraj et al., 2011, 2013; Sennett et al., 2011), and production of UDP-4-keto-xylose byproduct by UXS (Polizzi et al., 2012). Recent strategies to overcome these undesired outcomes during OPME UDP-Xyl production have introduced an additional three step enzymatic redox cascade and quinone reagent (Eixelsberger and Nidetzky, 2014), which may increase the cost to scale beyond the reported 5 mg purified yield.

An alternative natural pathway for stereopure UDP-Xyl production is possible through the arabinose salvage pathway (Figure 1). In plants, arabinose can be phosphorylated by a substrate specific sugar-1-kinase (arabinokinase), followed by addition of a nucleotide by a broad substrate UDP-sugar pyrophosphorylase (USP), to form UDP-Ara (Dolezal and Cobbett, 1991). UDP-Ara can be further epimerized at the C4 position to form stereopure UDP-Xyl, in a reaction that maintains cytosolic concentrations of both sugars for plant cell wall anabolism *in vivo* (Kotake et al., 2004). However, similar to the drawbacks of other *de novo* biosynthetic approaches, this strategy produces structurally related sugars that are notoriously difficult to separate using scalable purification techniques.

**Scheme 1:**
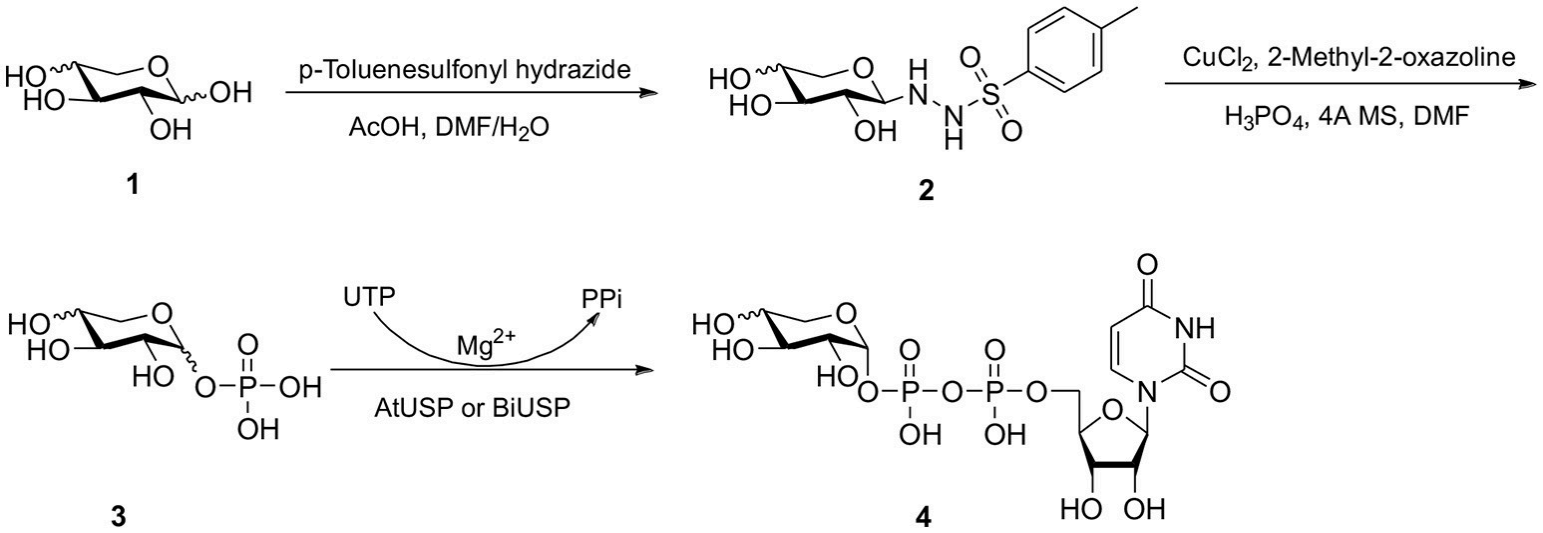
Chemo-enzymatic synthesis of UDP-α-D-xylose and UDP-β-L-arabinose.

Herein, we report an efficient chemoenzymatic method for producing stereopure UDP-α-D-xylose and UDP-β-L-arabinose (Scheme 1) that addresses reported obstacles to scalable production and separation. Our method: (1) Chemically converts monosaccharides to sugar-1-phosphate anomers, bypassing chemical protection steps, the more substrate specific kinases, and advanced purification techniques; (2) enzymatically performs asymmetric induction to exclusively generate the biologically relevant anomer, (3) avoids expensive redox cofactors and cascade enzymes, and (4) enables the production and purification of the structurally related UDP-α-D-xylose and UDP-β-L-arabinose epimers. The method has rapid applicability to the preparation of nucleotide sugars and may fill important gaps in leading enzymatic methods by mimicking the salvage pathway of nucleotide sugar biosynthesis in cases that a suitable kinase for a sugar or derivative is not available.

## Chemical synthesis of pentose-1-phosphate anomeric mixtures

For those monosaccharides which are not substrates for sugar kinases, chemical methods can be employed to produce the sugar-1-phosphates. We prepared xylose-1-phosphate and arabinose-1-phosphate separately from the corresponding monosaccharides in two steps (see Supplementary Methods) following the procedure reported by Edgar et al. (2012). Briefly, free sugars and p-toluenesulfonyl hydrazine (TSH) were reacted to generate glycosylsulfonylhydrazide adducts. These were further oxidized by anhydrous CuCl_2_ in the presence 2-methyl-2-oxazoline and an excess of crystalline phosphoric acid to give mixed anomers of the corresponding sugar-1-phosphates. Reaction mixtures were precipitated from dichloromethane (DCM), and solids were simply collected and extracted with water. Extractions were then treated dropwise with barium hydroxide to remove excess phosphate as the insoluble barium salt (Wood, 1968). Barium salts of sugar-1-phosphates remain soluble in water and can be precipitated from ethanol to provide convenient separation. However, the barium salts of the products are not biologically useful due to the low solubility of the compounds and possible effects of the counter-ion on the conformation and activity of enzymes. The precipitates were collected again, dissolved in water, and treated with 1M Na_2_CO_3_ to precipitate barium in its carbonate form, giving D-xylose-1-phosphate or L-arabinose-1-phosphate as disodium salts.

**Figure 2:**
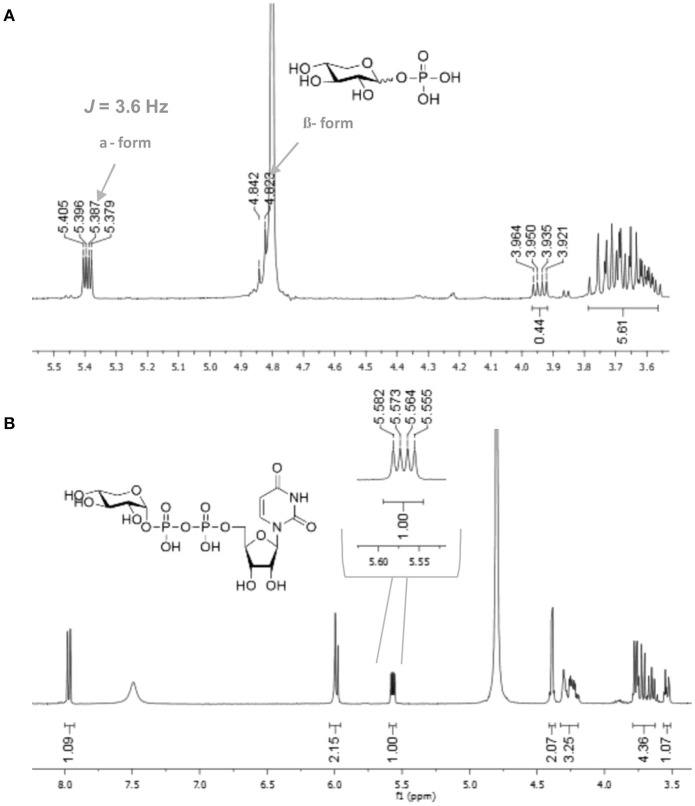
^1^H NMR of **(A)** xylose-1-phosphate and **(B)** UDP-α-D-xylose showing the high selectivity of enzyme for the α anomer.

In order to determine the anomeric configuration of each sugar-1-phosphate, we performed NMR analyses to estimate the relative α and β proton signals. Taking D-xylose-1-phosphate for example, we observed that both α and β proton signals were present in ^1^H NMR experiments (Figure 2A). The α anomer showed a chemical shift at 5.39 ppm with the coupling constant *J* = 3.4 Hz. The β anomer appeared at 4.83 ppm with higher coupling constant *J* = 7.6 Hz, although the β proton was not fully resolved from the solvent spectrum. Therefore, we roughly estimated the α/β anomer ratio using ^13^C NMR, which revealed a 2.1:1 proportion, respectively (see Supporting Information). NMR analysis of the L-arabinose-1-phosphate sample also revealed an anomeric mixture (α/β: 1/5.7; see Supporting Information).

Our results are consistent with Edgar et al. (2012), who reported an enhancement of glycosyl-1-phosphate anomers bearing phosphate in the axial orientation. Although we report an enhancement of β-L-arabinose-1-phosphate, inspection of the α-D-xylose-1-phosphate and β-L-arabinose-1-phosphate structures shows both anomeric phosphate groups are in the same axial orientation (Figure 1). By convention, it is the relative configurations between the anomeric and C-4 of pentoses that determine the assignment of the alpha or beta anomer. Of more importance for our aims, we observe that both D-xylose-1-phosphate and L-arabinose-1-phosphate were predominantly composed of the desired, biologically active anomers.

## Enzymatic selection for stereopure UDP-pentoses

In previous work, our lab has characterized USPs from *Arabidopsis thaliana* (AtUSP) and *Bifidobacterium infantis ATCC15697* (BiUSP) and found significant activity toward a broad range of substrates (Liu et al., 2013; Guo et al., 2015). In this study, AtUSP was codon optimized and cloned into pQE80L vector following our previous procedure. AtUSP and BiUSP were expressed in soluble form with His6 tag and purified by one step Ni-NTA affinity chromatography. Both enzymes were purified to approximately 90% purity by SDS-PAGE (Figure S1), with yields of 20 mg AtUSP/L and 10 mg BiUSP/L of LB culture. Approximate molecular weights of 70 and 60 KDa were observed, respectively, consistent with theoretical values.

With enzymes in hand, reactions containing anomeric mixtures of the corresponding sugar-1-phosphate substrates, AtUSP or BiUSP, MgCl_2_, uridine triphosphate (UTP), and Tris-HCl were incubated at 37°C overnight. Based on an initial sample of 10 mg of α/β-D-xylose-1-phosphate, we observed a good conversion to UDP-α-D-xylose in the presence of AtUSP, which was detected by thin layer chromatography (TLC) plates and purified with Bio-gel P-2 gel chromatography. After having established the feasibility of the preparation on small scale, a larger scale of 100 mg D-xylose-1-phosphate was conducted under the same conditions to provide 88 mg of the stereopure UDP-α-D-xylose with 45% yield (Table 1). Based on the anomeric ratio of the α/β-D-xylose-1-phosphate mixture, the yield was approximately 66%. The ^1^H-NMR spectrum of the UDP-α-D-xylose sample shows double-double peaks with the coupling constant (*J* = 3.6 Hz) at the chemical shift of 5.56 ppm (Figure 2B), corresponding to the anomeric proton in the alpha configuration. No peaks were found corresponding to a beta configuration in the final product, demonstrating the stereospecificity of the enzyme and its asymmetric induction effect (i.e., the preferential formation of one enantiomer or diastereoisomer over the other in a chemical reaction). In contrast to the AtUSP reaction, we did not observe any conversion of α/β-D-xylose-1-phosphate to UDP-xylose in the presence of BiUSP.

**Table 1:** Substrate specificity of AtUSP and BiUSP.

**Substrate**	**Sugar-1–P(α/β)^[a]^**	**Enzyme**	**Product**	**Yield% (mass)^[b]^**
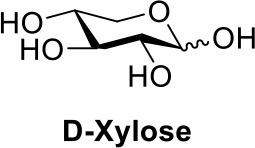	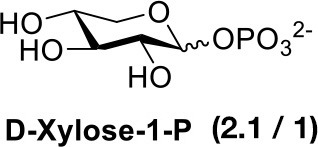	**AtUSP**	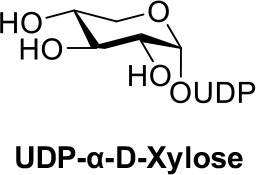	45% (88 mg)
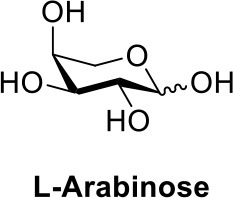	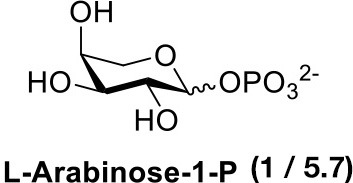	AtUSP BiUSP	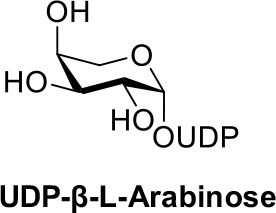	39% (76 mg) 49% (48 mg)

[a] *The ratios were determined by ^13^C NMR*;

[b] *Yields were calculated after purification*.

Following a similar procedure for the α/β-L-arabinose-1-phosphate sample, we observed the formation of UDP-β-L-arabinose in the presence of either AtUSP or BiUSP. AtUSP converted 100mg of α/β-L-arabinose-1-phosphate to 76 mg UDP-β-L-arabinose, and BiUSP converted 50 mg starting material to 48 mg product, generating yields of 39 and 49%, respectively (Table 1), without UDP-α-L-arabinose contamination (see Supporting Information).

Our experiment demonstrates an expedient method of general preparation that may be applied to other substrates at increased scale, especially for those monosaccharide substrates (e.g., D-xylose) which lack corresponding kinases. The initial two steps can provide various sugar-1-phosphate derivatives by utilizing the versatility and non-specificity of chemical synthesis. These two steps also circumvent traditional challenges of chemical synthesis by eliminating the need for protecting groups. However, the synthesis of sugar-1-phosphates produced α/β mixtures that are generally accepted as difficult to separate. Fortunately, the natural stereo-selectivity of UDP-sugar pyrophosphorylases can exclusively furnish the biologically relevant anomer of a UDP-sugar in good yield from a mixture of sugar-1-phosphates. Separation of unreacted sugar-1-phosphate can be achieved through a single exclusion (BioGel P2, Bio-Red) chromatography step, due to the difference in molecular weight compared to the corresponding UDP-sugar.

## Conclusion

The production of nucleotide sugars has long been a challenge to the study of complex carbohydrates. The chemo-enzymatic approach provides a number of advantages, as the versatility available to chemical synthesis is well-complemented by the efficiency and stereospecificity of enzymes. The helpful synergy between chemical and enzymatic steps in our procedure extends the application of leading biosynthetic methods to nucleotide sugars outside of the substrate scope of multi-enzyme systems, while providing a more facile approach to stereopure products than traditional chemical methods. The newly developed, three-step chemoenzymatic method is an efficient means of producing UDP-α-D-xylose, and UDP-β-L-arabinose on an improved scale. Furthermore, our experiment demonstrates an expedient method of general preparation with wide implications for other substrates, including non-natural sugar derivatives, for which a corresponding kinase has not been identified.

A recent review of nucleotide sugar production by Yu and Chen (2016) highlighted the use of OPME reactions to produce diverse UDP-sugars and oligosaccharides. However, the production of UDP-Xyl was noticeably less defined than systems for UDP-hexose sugars, and UDP-Ara production was absent. Further, while OPME systems were also reviewed in terms of coupled reactions with GTs, UDP-Xyl, and UDP-Ara applications were not discussed. One possible reason for the disparity in descriptions may be that UDP-Xyl and UDP-Ara have traditionally not fallen into the OPME salvage pathway scheme of sugar kinase and nucleotidyltransferase that can be easily coupled with a downstream GT.

In our current study, our improved chemo-enzymatic method has positive implications for OPME systems and GT-based technologies. We have shown that an expedient chemical synthesis of xylose-1-phosphate or arabinose-1-phosphate anomeric mixtures could be used as starting material for OPME systems including AtUSP and/or BiUSP for nucleotide sugar production. The resulting UDP-α-D-xylose or UDP-β-L-arabinose products are the naturally occurring anomers of each nucleotide sugar (Kotake et al., 2009), and anticipated to be substrates for classical, Leloir-type GTs (Coutinho et al., 2003). For example, UDP-Xyl is a substrate for multiple GTs: XT-I in the glycosylation of proteoglycan core proteins (Schon et al., 2006), SunS with sublancin antimicrobial glycopeptide (Oman et al., 2011), and an OleD triple mutant with coumarins (Williams et al., 2007). Similarly, UDP-Ara is the recognized donor molecule for multiple GTs: RRA3 (Velasquez et al., 2011) and EXG113 (Gille et al., 2009) in the glycosylation of O-linked extensins, and AtUGT78D3 with flavonoids (Kim et al., 2013). Ultimately, the ability to produce UDP-Xyl or UDP-Ara *in vitro* for purification, or *in situ* for coupling with GTs, may expand the experimental space available in glycan production and glycoengineering efforts.

## Author contributions

JW carried out chemo-enzymatic work and manuscript writing; HG performed chemical synthesis, characterization, and manuscript writing; SL performed enzyme work; MW carried out chemical synthesis; SP contributed manuscript writing and revision; PW designed and managed the study. All authors listed have made substantial, direct, and intellectual contributions to the work, and approved it for publication.

### Conflict of interest statement

SP and HG were employed by Chemily LLC, a company selling nucleotide sugars. The other authors declare that the research was conducted in the absence of any commercial or financial relationships that could be construed as a potential conflict of interest.

## References

[B1] Beahm B. J. Dehnert K. W. Derr N. L. Kuhn J. Eberhart J. K. Spillmann D.. (2014). A visualizable chain-terminating inhibitor of glycosaminoglycan biosynthesis in developing Zebrafish. Angew. Chem. Int. Edn. 53, 3347–3352. 10.1002/anie.201310569 24554559 PMC4029504

[B2] Chen Y. Dong W. Tan L. Held M. A. Kieliszewski M. J. (2015). Arabinosylation plays a crucial role in extensin cross-linking *in vitro*. Biochem. Insights 8, 1–13. 10.4137/BCI.S31353 26568683 PMC4629521

[B3] Coutinho P. M. Deleury E. Davies G. J. Henrissat B. (2003). An evolving hierarchical family classification for glycosyltransferases. J. Mol. Biol. 328, 307–317. 10.1016/S0022-2836(03)00307-3 12691742

[B4] Damerow S. Lamerz A.-C. Haselhorst T. Führing J. Zarnovican P. von Itzstein M.. (2010). Leishmania UDP-sugar pyrophosphorylase: the missing link in galactose salvage? J. Biol. Chem. 285, 878–887. 10.1074/jbc.M109.067223 19906649 PMC2801289

[B5] Dicker M. Tschofen M. Maresch D. König J. Juarez P. Orzaez D.. (2016). Transient glyco-engineering to produce recombinant IgA1 with defined N- and O-Glycans in plants. Front. Plant Sci. 7:18. 10.3389/fpls.2016.00018 26858738 PMC4731523

[B6] Dolezal O. Cobbett C. S. (1991). Arabinose kinase-deficient mutant of *Arabidopsis thaliana*. Plant Physiol. 96, 1255–1260. 16668327 10.1104/pp.96.4.1255 PMC1080923

[B7] Edgar L. J. Dasgupta S. Nitz M. (2012). Protecting-group-free synthesis of glycosyl 1-phosphates. Organ. Lett. 14, 4226–4229. 10.1021/ol3019083 22846058

[B8] Eixelsberger T. Nidetzky B. (2014). Enzymatic redox cascade for one-pot synthesis of uridine 5′-Diphosphate xylose from uridine 5′-diphosphate glucose. IUBMB Life 356, 3575–3584. 10.1002/adsc.201400766 26190959 PMC4498474

[B9] Ernst C. Klaffke W. (2003). Chemical synthesis of uridine diphospho-D-xylose and UDP-L-arabinose. J. Organ. Chem. 68, 5780–5783. 10.1021/jo034379u 12839484

[B10] Errey J. C. Mukhopadhyay B. Kartha K. P. Field R. A. (2004). Flexible enzymatic and chemo-enzymatic approaches to a broad range of uridine-diphospho-sugars. Chem. Commun. 23, 2706–2707. 10.1039/b410184g 15568077

[B11] Esko J. D. Kimata K. Lindahl U. (2009). Proteoglycans and sulfated glycosaminoglycans, in Essentials of Glycobiology, 2nd Edn, eds Varki, A. Cummings, R. D., and Esko J. D. Cold Spring Harbor, NY: Cold Spring Harbor Laboratory Press. 20301236

[B12] Geserick C. Tenhaken R. (2013). UDP-sugar pyrophosphorylase is essential for arabinose and xylose recycling, and is required during vegetative and reproductive growth in Arabidopsis. Plant J. 74, 239–247. 10.1111/tpj.12116 23373795 PMC3659416

[B13] Gille S. Hänsel U. Ziemann M. Pauly M. (2009). Identification of plant cell wall mutants by means of a forward chemical genetic approach using hydrolases. Proc. Natl. Acad. Sci. U.S.A. 106, 14699–14704. 10.1073/pnas.0905434106 19667208 PMC2731844

[B14] Gu X. Lee S. G. Bar-Peled M. (2011). Biosynthesis of UDP-xylose and UDP-arabinose in Sinorhizobium meliloti 1021: first characterization of a bacterial UDP-xylose synthase, and UDP-xylose 4-epimerase. Microbiology 157(Pt 1), 260–269. 10.1099/mic.0.040758-0 20847005 PMC3068629

[B15] Guan W. Cai L. Fang J. Wu B. Wang P. G. (2009). Enzymatic synthesis of UDP-GlcNAc/UDP-GalNAc analogs using N-acetylglucosamine 1-phosphate uridyltransferase (GlmU). Chem. Commun. 45, 6976–6978. 10.1039/b917573c 19904366

[B16] Guo Y. Fang J. Li T. Li X. Ma C. Wang X.. (2015). Comparing substrate specificity of two UDP-sugar pyrophosphorylases and efficient one-pot enzymatic synthesis of UDP-GlcA and UDP-GalA. Carbohydr. Res. 411, 1–5. 10.1016/j.carres.2015.04.001 25942062 PMC4481193

[B17] Harper A. D. Bar-Peled M. (2002). Biosynthesis of UDP-xylose. Cloning and characterization of a novel Arabidopsis gene family, UXS, encoding soluble and putative membrane-bound UDP-glucuronic acid decarboxylase isoforms. Plant Physiol. 130, 2188–2198. 10.1104/pp.009654 12481102 PMC166730

[B18] Ishimizu T. Uchida T. Sano K. Hase S. (2005). Chemical synthesis of uridine 5′-diphospho-α-D-xylopyranose. Tetrahedron Asymmet. 16, 309–311. 10.1016/j.tetasy.2004.11.073

[B19] Kadirvelraj R. Sennett N. C. Custer G. S. Phillips R. S. Wood Z. A. (2013). Hysteresis and negative cooperativity in human UDP-glucose dehydrogenase. Biochemistry 52, 1456–1465. 10.1021/bi301593c 23363239

[B20] Kadirvelraj R. Sennett N. C. Polizzi S. J. Weitzel S. Wood Z. A. (2011). Role of packing defects in the evolution of allostery and induced fit in human UDP-glucose dehydrogenase. Biochemistry 50, 5780–5789. 10.1021/bi2005637 21595445

[B21] Kim H. S. Kim B.-G. Sung S. Kim M. Mok H. Chong Y.. (2013). Engineering flavonoid glycosyltransferases for enhanced catalytic efficiency and extended sugar-donor selectivity. Planta 238, 683–693. 10.1007/s00425-013-1922-0 23801300

[B22] Kotake T. Takata R. Verma R. Takaba M. Yamaguchi D. Orita T.. (2009). Bifunctional cytosolic UDP-glucose 4-epimerases catalyse the interconversion between UDP-D-xylose and UDP-L-arabinose in plants. Biochem. J. 424, 169–177. 10.1042/BJ20091025 19754426

[B23] Kotake T. Yamaguchi D. Ohzono H. Hojo S. Tsumuraya Y. Kaneko S.. (2004). UDP-sugar pyrophosphorylase with broad substrate specificity toward various monosaccharide 1-phosphates from pea sprouts. J. Biol. Chem. 279, 45728–45736. 10.1074/jbc.M408716200 15326166

[B24] Lee T. V. Leonardi J. Jafar-Nejad H. Sethi M. K. Buettner F. F. Bakker H.. (2013). Negative regulation of notch signaling by Xylose. PLoS Genet. 9:e1003547. 10.1371/journal.pgen.1003547 23754965 PMC3675014

[B25] Li L. Liu Y. Wan Y. Li Y. Chen X. Zhao W.. (2013). Efficient Enzymatic synthesis of guanosine 5 '-Diphosphate-sugars and derivatives. Organ. Lett. 15, 5528–5530. 10.1021/ol402585c 24117142 PMC3915774

[B26] Li L. Liu Y. Li T. Wang W. Yu Z. Ma C.. (2015). Efficient chemoenzymatic synthesis of novel galacto-N-biose derivatives and their sialylated forms. Chem. Commun. 51, 10310–10313. 10.1039/C5CC03746H 26023910 PMC4498953

[B27] Liu J. Zou Y. Guan W. Zhai Y. Xue M. Jin L.. (2013). Biosynthesis of nucleotide sugars by a promiscuous UDP-sugar pyrophosphorylase from Arabidopsis thaliana (AtUSP). Bioorg. Med. Chem. Lett. 23, 3764–3768. 10.1016/j.bmcl.2013.04.090 23707255

[B28] Muthana M. M. Qu J. Li Y. Zhang L. Yu H. Ding L.. (2012). Efficient one-pot multienzyme synthesis of UDP-sugars using a promiscuous UDP-sugar pyrophosphorylase from Bifidobacterium longum (BLUSP). Chem. Commun. 48, 2728–2730. 10.1039/c2cc17577k 22306833

[B29] Muthana M. M. Qu J. Xue M. Klyuchnik T. Siu A. Li Y.. (2015). Improved one-pot multienzyme (OPME) systems for synthesizing UDP-uronic acids and glucuronides. Chem Commun (Camb). 51, 4595–4598. 10.1039/c4cc10306h 25686901 PMC4348237

[B30] Oman T. J. Boettcher J. M. Wang H. Okalibe X. N. van der Donk W. A. (2011). Sublancin is not a lantibiotic but an S-linked glycopeptide. Nat. Chem. Biol. 7:78 10.1038/nchembio.509 21196935 PMC3060661

[B31] Pauly M. Porchia A. Olsen C. E. Nunan K. J. Scheller H. V. (2000). Enzymatic synthesis and purification of uridine Diphospho-β-l-arabinopyranose, a substrate for the biosynthesis of plant polysaccharides. Anal. Biochem. 278, 69–73. 10.1006/abio.1999.4411 10640355

[B32] Polizzi S. J. Walsh R. M. Jr. Peeples W. B. Lim J. M. Wells L. Wood Z. A. (2012). Human UDP-α-D-xylose synthase and *E. coli* ArnA conserve a conformational shunt that controls whether xylose or 4-keto-xylose is produced. Biochemistry 51, 8844–8855. 10.1021/bi301135b 23072385 PMC4932848

[B33] Roseman S. Distler J. J. Moffatt J. G. Khorana H. G. (1961). Nucleoside polyphosphates. XI. An improved general method for the synthesis of nucleotide coenzymes. Syntheses of Uridine-5′, Cytidine-5′ and Guanosine-5′ Diphosphate derivatives. J. Am. Chem. Soc. 83, 659–663. 10.1021/ja01464a035

[B34] Schön S. Prante C. Bahr C. Kuhn J. Kleesiek K. Götting C. (2006). Cloning and recombinant expression of active full-length xylosyltransferase I (XT-I) and characterization of subcellular localization of XT-I and XT-II. J. Biol. Chem. 281, 14224–14231. 10.1074/jbc.M510690200 16569644

[B35] Sennett N. C. Kadirvelraj R. Wood Z. A. (2011). Conformational flexibility in the allosteric regulation of human UDP-alpha-D-glucose 6-dehydrogenase. Biochemistry 50, 9651–9663. 10.1021/bi201381e 21961565

[B36] Sim M. M. Kondo H. Wong C. H. (1993). Synthesis and use of glycosyl phosphites: an effective route to glycosyl phosphates, sugar nucleotides, and glycosides. J. Am. Chem. Soc. 115, 2260–2267. 10.1021/ja00059a023

[B37] Velasquez S. M. Ricardi M. M. Dorosz J. G. Fernandez P. V. Nadra A. D. Pol-Fachin L.. (2011). O-Glycosylated cell wall proteins are essential in root hair growth. Science 332, 1401–1403. 10.1126/science.1206657 21680836

[B38] Wagner G. K. Pesnot T. Field R. A. (2009). A survey of chemical methods for sugar-nucleotide synthesis. Nat. Prod. Rep. 26, 1172–1194. 10.1039/b909621n 19693414

[B39] Williams G. J. Zhang C. Thorson J. S. (2007). Expanding the promiscuity of a natural-product glycosyltransferase by directed evolution. Nat. Chem. Biol. 3:657. 10.1038/nchembio.2007.28 17828251

[B40] Wolf S. Berrio R. M. Meier C. (2011). Synthesis of nonnatural nucleoside diphosphate sugars. Eur. J. Organ. Chem. 31, 6304–6313. 10.1002/ejoc.201100906

[B41] Wood T. (1968). The detection and identification of intermediates of the pentose phosphate cycle and related compounds. J. Chromatogr. A 35, 352–361. 10.1016/S0021-9673(01)82396-7 4298075

[B42] Yang T. Bar-Peled M. (2010). Identification of a novel UDP-sugar pyrophosphorylase with a broad substrate specificity in *Trypanosoma cruzi*. Biochem. J. 429, 533–543. 10.1042/BJ20100238 20482518

[B43] Yu H. Chen X. (2016). One-pot multienzyme (OPME) systems for chemoenzymatic synthesis of carbohydrates. Org. Biomol. Chem. 14, 2809–2818. 10.1039/C6OB00058D 26881499 PMC4795158

[B44] Zhang Q. Liu H. W. (2000). Studies of UDP-Galactopyranose Mutase from *Escherichia coli*: an unusual role of reduced FAD in its catalysis. J. Am. Chem. Soc. 122, 9065–9070. 10.1021/ja001333z

