# Supplementary material for: Facile and Stereo-Selective Synthesis of UDP-α-D-xylose and UDP-β-L-arabinose Using UDP-Sugar Pyrophosphorylase
Source: Front Chem. 2018 May 23;6:163. doi: 10.3389/fchem.2018.00163 (PMC5974040; doi:10.3389/fchem.2018.00163)

Facile and stereo-selective synthesis of UDP-α-D-xylose and UDP-β-L-arabinose using UDP-sugar pyrophosphorylase

Supporting information

JiaJia Wang[a,b], Harmon Greenway[b,c], Shanshan Li[b], Mohui Wei[b], Samuel J Polizzi [c], and Peng George Wang*[,b]

a. School of Basic Medical Science, Joint National Laboratory for Antibody Drug engineering, Henan University, Kaifeng, 475004, PR China

b. Department of Chemistry and Center for Diagnostics & Therapeutics, Georgia State University, Atlanta, GA, 30303, USA

c. Chemily, LLC 58 Edgewood Ave. NE, Atlanta, GA

* Corresponding author. Email: pwang11@gsu.edu

Table of Contents

1. Expression and Purification of pQE-80L AtUSP and BiUSP……………………………………………….S2
2. Thin-layer chromatography (TLC) of UDP-α-D-Xylose and UDP-β-L-Arabinose reactions ………..S2
3. General experiment methods……………………………………………………………………………………….S3
4. NMR and HRMS Spectra of synthesized compounds………………………………………………………S8

1. Expression and Purification of pQE-80L AtUSP and BiUSP

LB medium (10 g tryptone, 5 g yeast extract and 10 g NaCl per liter) containing 50 ug/mL kanamycin was inoculated with BL21 strain and grown at 37°C shaking at 180 rpm overnight. The culture was enlarged into 2 L medium until OD600nm reached 0.4 - 0.6, then induced with isopropyl beta-D-1-thiogalactopyranoside (IPTG) at a final concentration of 0.1 mM and grown at 16°C and 150 rpm overnight. Cells were harvested by centrifugation at 8,000g for 30 min and suspended in lysis buffer (10 mM Tris-HCI, pH 8, 150 mM NaCl, 5mM $MgCI_2$). The cells were disrupted by ultrasonication using a microtip with 20% power for 1h (3 sec on and 3 sec off) on the ice and applied to centrifugation (13,000g for 30 min). The supernatants of lysates were loaded onto a nickel affinity chromatography column, which was equilibrated with binding buffer (10 mM Tris-HCI, pH 8, 150 mM NaCl, 5mM $MgCI_2$). After washing unbound proteins with washing buffer (10 mM Tris-HCI, pH 8, 150 mM NaCl, 5mM $MgCI_2$, 20mM imidazole), the fusion proteins were eluted with elution buffer (10 mM Tris-HCI, pH 8, 150 mM NaCl, 5mM $MgCI_2$, 500mM imidazole). The purified protein was concentrated using a 10 kD Amicon Ultra Centrifugal Filter Unit (Millipore, Ireland), and the buffer was changed to the reaction buffer (10 mM Tris-HCI, pH 8, 150 mM NaCl, 5mM $MgCI_2$) to remove imidazole. Similar procedures were used to purify BiUSP. An approximate molecular weight of 70 KDa and 60 KDa were observed on SDS-PAGE (FigureS1, Lanes 1 and 2), which is consistent with theoretical values.

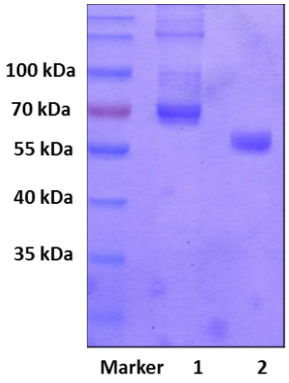

Figure S1. SDS-PAGE analysis of purified enzymes. Lanes: 1. AtUSP; 2. BiUSP.

2. Thin-layer chromatography (TLC)of UDP-α-D-Xylose and UDP-β-L-Arabinose reactions

For TLC analysis, 0.5 uL of each sample was directly spotted on silica gel plates, developed by Ethyl acetate/MeOH/CH_3COOH/H_2O = 5:3:1.5:0.5 (v/v/v/v),dried and visualized under 254 nm UV light.

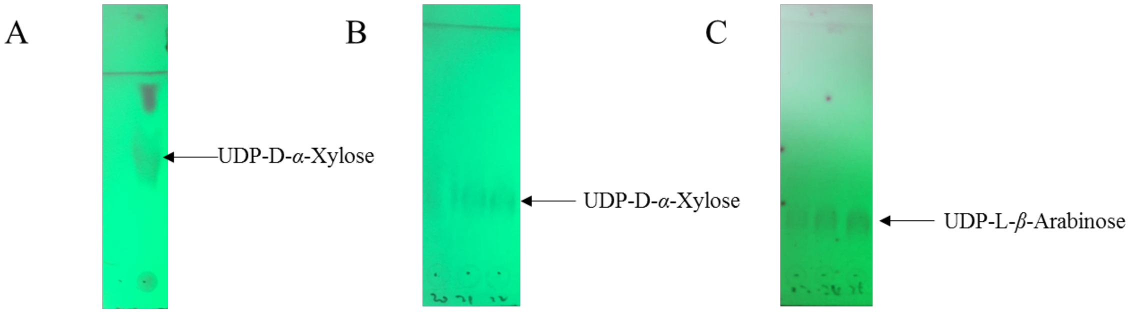

Figure S2. TLC analysis of UDP-α-D-Xylose and UDP-β-L-Arabinose before and after purification using Bio-gel P-2 gel chromatography. S2A: The reacted solution of UDP-α-D-Xylose after digestion with Calf intestinal alkaline phosphatase (CIP); S2B: Purified UDP-α-D-Xylose with P2 column; S2C: Purified UDP-β-L-Arabinose with P2 column from the reaction of 1-P-arabinose and AtUSP.

3. General methods

The α-D-Xylose and β-L-Arabinose starting materials were purchased from Carbosynth.2-Methyl-2-oxazoline, anhydrous cupric chloride, crystalline phosphoric acid and extra dry dimethylformamide were supplied from Sigma-Aldrich, and p-toluenesulfonyl hydrazide was purchased from Alfa Aesar. All purchased reagents were used without further purification. Reactions were monitored by TLC visualized under 254 nm UV light and/or stained with a solution of p-anisaldehyde solution (ethanol/p-anisaldehyde/acetic acid/sulfuric acid 135:5:4:1.5), followed by heating on a hot plate. ^{1}H NMR and ^{13}C NMR experiments were recorded with Bruker AV 400 spectrometer at 400 MHz (^{1}H NMR), 100 MHz (^{13}C NMR), 162 MHz (^{31}P NMR) using CD_3OD or D_2O as solvents. The chemical shifts were recorded in part per million (ppm) relative to TMS and the coupling constants *J* were reported in hertz (Hz). High-resolution electrospray-ionization mass spectra (HRMS-ESI) were obtained on a Thermo Orbitrap elite. Optical rotations were measured in water at 20 °C at 589 nm (Na), using a 10 cm x 1 mL cell on a Perkin Elmer Model 343 Polarimeter.

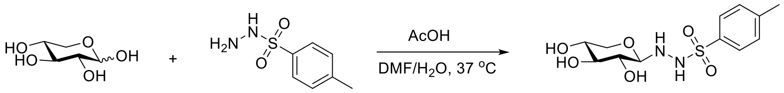

D-Xylose **TSH-D-Xylose**

Synthesis of TSH-D-Xylose:α-D-Xylose (10 g, 66.7 mmol) and p-toluenesulfonyl hydrazide (TSH) (13.0 g, 70 mmol) were suspended in DMF (25 mL) and distilled H_2O (12.5 mL) in a polypropylene tube (50 mL). Acetic acid (2.5 mL, 33.2 mmol) was then added and the mixture was incubated at 37 °C water bath without stirring until all solids had dissolved (~ 24 h). The clear reaction mixture was then poured into diethyl ether (500 mL) and stirred vigorously for 4 h. The supernatant was poured off. Fresh diethyl ether (500 mL) was added and the mixture was stirred overnight. The precipitate was collected via vacuum filtration to afford TSH-D-Xylose as white solid (20.5 g, 96.7%).^{1}H NMR (400 MHz, CD_3OD) δ 7.72 (d, *J* = 8.2 Hz, 2H, Ar-2H), 7.32 (d, *J* = 8.0 Hz, 2H,Ar-2H), 3.72 (dd, *J* = 11.3, 5.4 Hz, 1H, H-5a), 3.63 (d, *J* = 8.7 Hz, 1H, H-1), 3.38 – 3.34 (m,2H, H-2, H-4), 3.24 – 3.21 (m, 1H, 3-H), 3.01 (dd, *J* = 11.1, 10.6 Hz, 1H, H-5b), 2.36 (s, 3H, Ar-CH_3); ^{13}C NMR (100 MHz, CD_3OD) δ 145.15 (Ar), 137.11 (Ar), 130.58 (Ar), 129.08 (Ar), 92.36 (C-1), 78.13 (C-2), 71.37 (C-3), 71.23 (C-4), 68.32 (C-5), 21.49 (CH_3).

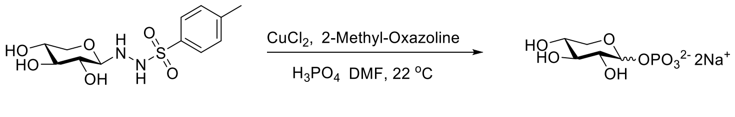

TSH-D-Xylose **D-Xylose-1-P**

Synthesis of D-Xylose-1-Phosphate:TSH-D-Xylose(5 g, 15.7 mmol), activated 4 Å molecular sieves (4 g) and a dry magnetic stir bar were put in a dry 250 mL round bottom flask under the protection of Nitrogen. Dry DMF (30 mL) was added to the flask and the mixture was stirred for 15 minutes under N_2(g) atmosphere at room temperature. Anhydrous cupric chloride (8.65 g, 64.4 mmol) was placed in another 250mL dry flask and dissolved in dry DMF (20 mL). 2-methyl-2-oxazoline (5.43 mL, 64.4 mmol) was added to the cupric chloride solution and the mixture was shaken vigorously for 1min. Crystalline phosphoric acid (24.6 g, 0.25 mol) was placed in a separate 250 mL dry flask, dissolved in dry DMF (20 mL) which was subsequently added to the cupric chloride-oxazoline solution. This mixture was shaken vigorously for 1 min and then added rapidly to the solution containing the TSH donor via syringe at room temperature. After stirring for 18 hours the crude reaction mixture was poured into dichloromethane (1000 mL) and the mixture was stirred for 1 h, the resultant precipitate was collected via vacuum filtration. The precipitate was dissolved in water (~100 mL) and any remaining precipitate was removed from the clear water phase via centrifugation. Then, the clear water phase was treated dropwise with an aqueous saturated solution of barium hydroxide until no additional precipitate formed. The resulting precipitate was removed by centrifugation. The pH of the supernatant was adjusted to 8 and concentrated to around 15 mL, which was separated into three 50 mL centrifuge tubes. Then re-precipitated via addition of absolute ethanol (~ 40 mL) and the precipitate was isolated via centrifugation. After that the precipitate was re-dissolved in 10 mL H_2O, and 1 M Na_2CO_3 was added dropwise to precipitate Ba^{2+}. The precipitate was removed via centrifugation, and the supernatant was concentrated and lyophilized to give disodium D-Xylose-1-Phosphate as a mixture of α/βanomers (1.97g, 46%).^{1}H NMR (400 MHz, D_2O) δ 5.39 (dd, *J* = 7.1, 3.4 Hz, 1H, H-1*α*), 3.94 (dd, *J* = 11.6, 5.4 Hz, 0.44H, H-5), 3.75 – 3.63 (m, 5.6H, H-3, H-4,), 3.46 (ddd, *J* = 5.2, 3.2, 1.6 Hz, 1.3H, H-5), 3.35 – 3.27 (m, 1H, H-2); ^{13}C NMR (100 MHz, D_2O) δ 97.74 (C-1α), 97.70 (C-1α),

93.69 (C-1β), 93.63 (C-1β), 75.55 (C-3), 74.22 (C-3), 73.31 (C-2), 72.21 (C-2), 72.14 (C-2), 69.56 (C-4), 69.21 (C-4), 65.34 (C-5), 61.54 (C-5); ^{31}P NMR (162 MHz, D_2O) δ 2.52, 2.35;

UDP-α-D-Xylose

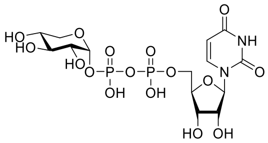

D-Xylose-1-phosphate (100 mg, 0.36 mmol), UTP (4 mL, 100 mM, 1.1 eq) and $MgCl_2$ (0.9 mL, 200 mM) were dissolved in water in50 mL centrifuge tube, and then Tris-HCl buffer (100 mM, pH = 8.0) was added to adjust the final pH 7-8. AtUSP (2 mg) and dH_2O were added to bring the final reaction volume to 18 mL, and then mixed by gentle shaking to make the mixture homogeneous. The reaction was carried out by incubating in thermostatic water bath at 37 ℃ overnight. The reaction was monitored by TLC (EtOAc: MeOH: H_2O: HOAc = 5:3:1:0.5 by volume) and additional enzyme (0.5 mg) was added to ensure a high level of Xylose-1-phosphate was converted. After the starting sugar-1-P was consumed completely, 1M NaOH was added to adjust the pH around 8~9, alkaline phosphatase was added to digest of the excess UTP. The digestion of nucleotides was detected with TLC until no obvious UTP remained. When the reaction finished, equivalent volume of ice-cold ethanol was added to quench the reaction and incubating at 4℃ for 2h. The precipitant was removed by centrifugation and the supernatant was collected and concentrated by vacuum pump to provide the crude product around 1-2 mL, which was purified by P-2 column (BioGel P2, Bio-Rad) for multiple times. Fractions containing products were collected and lyophilized to afford pure UDP-α-D-Xylose(88 mg, 45%)[1, 2].^{1}H NMR (400 MHz, D_2O) δ 7.97 (d, *J* = 8.4 Hz, 1H, 6''-H), 5.99 (m, 2H, 5''-H), 5.57 (dd, *J* = 7.3, 3.6 Hz, 1H, 1-H), 4.40 – 4.37 (m, 2H, 2'-H, 3'-H), 4.30 – 4.21 (m, 3H, 5'-H, 4'-H, 3'-H), 3.78 -3.62 (m, 4H, 2-H, 3-H, 4-H, 5-H), 3.64 - 3.61 (m, 1H, 5-H); ^{13}C NMR (100 MHz, D_2O) δ 166.22 (C-4''), 151.80 (C-2''), 141.60 (C-6''), 102.64 (C-5''), 95.68 (C-1, 95.62 (C-1), 88.37 (C-1'), 83.26 (C-4'), 73.76 (C-3'), 73.02 (C-2'), 71.66 (C-4), 69.62 (C-3), 69.16 (C-2), 64.93 (C-5'), 64.88 (C-5'), 62.23 (C-5).^{31}P NMR (162 MHz, D_2O) δ -11.41 (d, *J* = 20.3 Hz), -13.13 (d, *J* = 20.3 Hz);HRMS (ESI) Calculated for [$C_{14}H_{21}N_2O_{16}P_2 - H^+$] 535.0366, found 535.0324; [α_D^{20}] +13.0 (*c*0.67, H_2O)

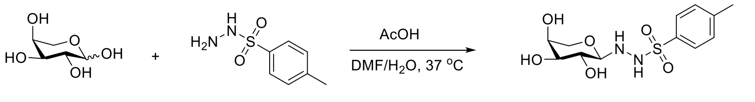

Synthesis of TSH-L-Arabinose:β-L-Arabinose (10 g, 66.7 mmol) and p-toluenesulfonyl hydrazide (TSH) (13.0 g, 70 mmol) were suspended in DMF (25 mL) and distilled H_2O (12.5 mL) in a polypropylene tube

(50 mL). Acetic acid (2.5 mL, 33.2 mmol) was then added and the mixture was incubated at 37 °C water bath without stirring until all solids had dissolved (~ 24 h). The clear reaction mixture was then poured into diethyl ether (500 mL) and stirred vigorously for 4 h. The supernatant was poured off. Fresh diethyl ether (500 mL) was added and the mixture was stirred overnight. The precipitate was collected via vacuum filtration to afford TSH-L-Arabinose as white solid (20.2 g, 95.3%). ^{1}H NMR (400 MHz, CD_3OD) δ 7.79 (d, *J* = 8.3 Hz, 2H, Ar-2H), 7.38 (d, *J* = 8.0 Hz, 2H, Ar-2H), 3.81 – 3.69 (m, 3H, H-1, H-3, H-5a), 3.52 – 3.49 (m, 1H, H-4), 3.46 (dd, *J* = 13.2, 2.3 Hz, 1H, H-5b),3.32 – 3.30 (m, 1H, H-2) 2.43 (s,3H, Ar-CH_3); ^{13}C NMR (100 MHz, CD_3OD) δ 145.12 (Ar), 137.32 (Ar), 130.58 (Ar), 129.09(Ar), 92.52 (C-1), 74.43 (C-3), 70.00 (C-2), 69.32 (C-4), 68.14 (C-5), 21.49 (CH_3).

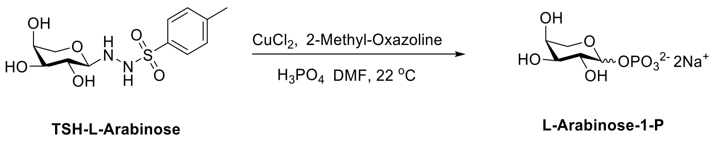

Synthesis of L-Arabinose-1-Phosphate:TSH-L-Arabinose (5 g, 15.7 mmol), activated 4 Å molecular sieves (4 g) and a dry magnetic stir bar were put in a dry 250 mL round bottom flask under the protection of Nitrogen. Dry DMF (30 mL) was added to the flask and the mixture was stirred for 15 minutes under N_2 (g) atmosphere at room temperature. Anhydrous cupric chloride (8.65 g, 64.4 mmol) was placed in another 250mL dry flask and dissolved in dry DMF (20 mL). 2-methyl-2-oxazoline (5.43 mL, 64.4 mmol) was added to the cupric chloride solution and the mixture was shaken vigorously for 1min. Crystalline phosphoric acid (24.6 g, 0.25 mol) was placed in a separate 250 mL dry flask, dissolved in dry DMF (20 mL) which was subsequently added to the cupric chloride-oxazoline solution. This mixture was shaken vigorously for 1 min and then added rapidly to the solution containing the TSH donor via syringe at room temperature. After stirring for 18 hours the crude reaction mixture was poured into dichloromethane (1000 mL) and the mixture was stirred for 1 h, the resultant precipitate was collected via vacuum filtration. The precipitate was dissolved in water (~100 mL) and any remaining precipitate was removed from the clear water phase via centrifugation. Then, the clear water phase was treated dropwise with an aqueous saturated solution of barium hydroxide until no additional precipitate formed. The resulting precipitate were removed by centrifugation. The pH of the supernatant was adjusted to 8 and concentrated to around 15 mL, which was separated into three 50 mL centrifuge tubes. Then re-precipitated via addition of absolute ethanol (~ 40 mL) and the precipitate was isolated via centrifugation. After that the precipitate was re-dissolved in 10 mL H_2O, and 1 M Na_2CO_3 was added dropwise to precipitate Ba^{2+}. The precipitate was removed via centrifugation, and the supernatant was concentrated and lyophilized to give disodium L-Arabinose-1-Phosphate as white solid (2.39 g, 55.7%).^{1}H NMR (400 MHz, D_2O) δ 5.37 (dd, *J* = 6.8, 3.4 Hz, 1H, H-1β), 3.95 (d, *J* = 12.8 Hz, 1H, H-5), 3.91 – 3.87 (m, 1H, H-3), 3.79 (dd, *J* = 10.0, 3.4 Hz, 1H, H-4), 3.70 – 3.66 (m, 1H, H-5), 3.54 (dd, *J* = 12.8, 2.1 Hz, 1H); ^{13}C NMR (100 MHz, D_2O) δ 94.69(C-1), 94.63 (C-1), 68.80 (C-3), 68.69 (C-3), 68.55 (C-2), 68.47 (C-2), 63.30 (C-4), 59.26 (C-5); ^{31}P NMR (162 MHz, D_2O) δ 0.75.

UDP-β-L-Arabinose

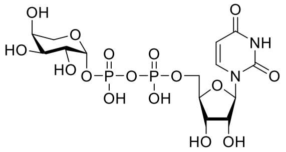

L-Arabinose-1-phosphate (100 mg, 0.36 mmol), UTP (4 mL, 100 mM, 1.1 eq) and $MgCl_2$ (0.9 mL, 200 mM) were dissolved in water in 50 mL centrifuge tube, and then Tris-HCl buffer (100 mM, pH = 8.0) was added to adjust the final pH 7-8.AtUSP (3 mg) and dH_2O were added to bring the final reaction volume to 18 mL, and then mixed gently to make the mixture homogeneous. The reaction was carried out by incubating in thermostatic water bath at 37 ℃ overnight. The reaction was monitored by TLC (EtOAc: MeOH: H_2O: HOAc = 5:3:1:0.5 by volume) and additional enzyme (1.2 mg) was added to ensure a high level of Arabinose-1-phosphate was converted. After the starting sugar-1-P was consumed completely, 1M NaOH was added to adjust the pH around 8~9, alkaline phosphatase was added to digest of the excess UTP. The digestion of nucleotides was detected with TLC until no obvious UTP remained. When the reaction finished, an equivalent volume of ice-cold ethanol was added to quench the reaction and incubating at 4 ℃ for 2h. The precipitant was removed by centrifugation and the supernatant was collected and concentrated by vacuum pump to provide the crude product around 1-2 mL, which was purified by P-2 column (BioGel P2, Bio-Rad) for multiple times, fractions containing products were collected and lyophilized to afford UDP-L-β-arabinose (76 mg, 39%)[3].

L-Arabinose-1-phosphate (50 mg, 0.18 mmol) was treated with the similar procedure as above in the presence of another UDP-sugar pyrophosphorylase, BiUSP(1mg), to produce UDP-β-L-arabinose (48 mg, 49%).^{1}H NMR (400 MHz, D_2O) δ 7.97 (d, J = 8.0 Hz, 1H, 6''-H), 6.00 – 5.98 (m, 2H, 5''-H, 1'-H), 5.62 (dd, J = 7.2, 3.6 Hz, 1H, 1-H), 4.41 – 4.37 (m, 2H, 2'-H, 3'-H), 4.32 – 4.22 (m, 3H, 5'-2H, 4'-H), 4.14 (d, J = 12.8 Hz, 1H, 5-H), 4.06 – 4.03 (m, 1H, 4-H), 3.94 (dd, J = 10.0, 3.2 Hz, 1H, 3-H), 3.83 (dt, J = 10.0, 3.2 Hz, 1H, 2-H), 3.74 (dd, J = 12.8, 2.0 Hz, 1H, 5H); ^{13}C NMR (100 MHz, D_2O) δ 166.33 (C-4''), 151.90 (C-2''), 141.59 (C-6''), 102.65 (C-5''), 96.23 (C-1), 96.16 (C-1), 88.37 (C-1'), 83.18 (C-4'), 73.75 (C-3'), 69.64 (C-3'), 68.85 (C-2'), 68.70 (C-4), 68.42 (C-3), 68.33 (C-2), 64.93 (C-5'), 64.87 (C-5'), 63.96 (C-5); ^{31}P NMR (162 MHz, D_2O) δ -11.20 (d, J = 20.5 Hz), -12.70 (d, J = 20.5 Hz); HRMS (ESI) Calculated for $[C_{14}H_{21}N_2O_{16}P_2 - H^+]$ 535.0366, found 535.0329;$[\alpha_D^{20}]$ +36.2 (c 1.0, H_2O).

Reference

1. Bar-Peled, M., C.L. Griffith, and T.L. Doering, *Functional cloning and characterization of a UDP- glucuronic acid decarboxylase: the pathogenic fungus Cryptococcus neoformans elucidates UDP-xylose synthesis.* Proc Natl Acad Sci U S A, 2001. **98**(21): p. 12003-8.
2. Rosenberger, A.F.N., et al., *UDP-xylose and UDP-galactose synthesis in Trichomonas vaginalis.* Molecular and Biochemical Parasitology, 2012. **181**(1): p. 53-56.
3. Zhang, Q. and H.W. Liu, *Chemical synthesis of UDP-beta-L-arabinofuranose and its turnover to UDP-beta-L-arabinopyranose by UDP-galactopyranose mutase.* Bioorg Med Chem Lett, 2001. **11**(2): p. 145-9.

4. NMR Spectra of synthesized compounds

^{1}H NMR and ^{13}C NMR of TSH-D-Xylose

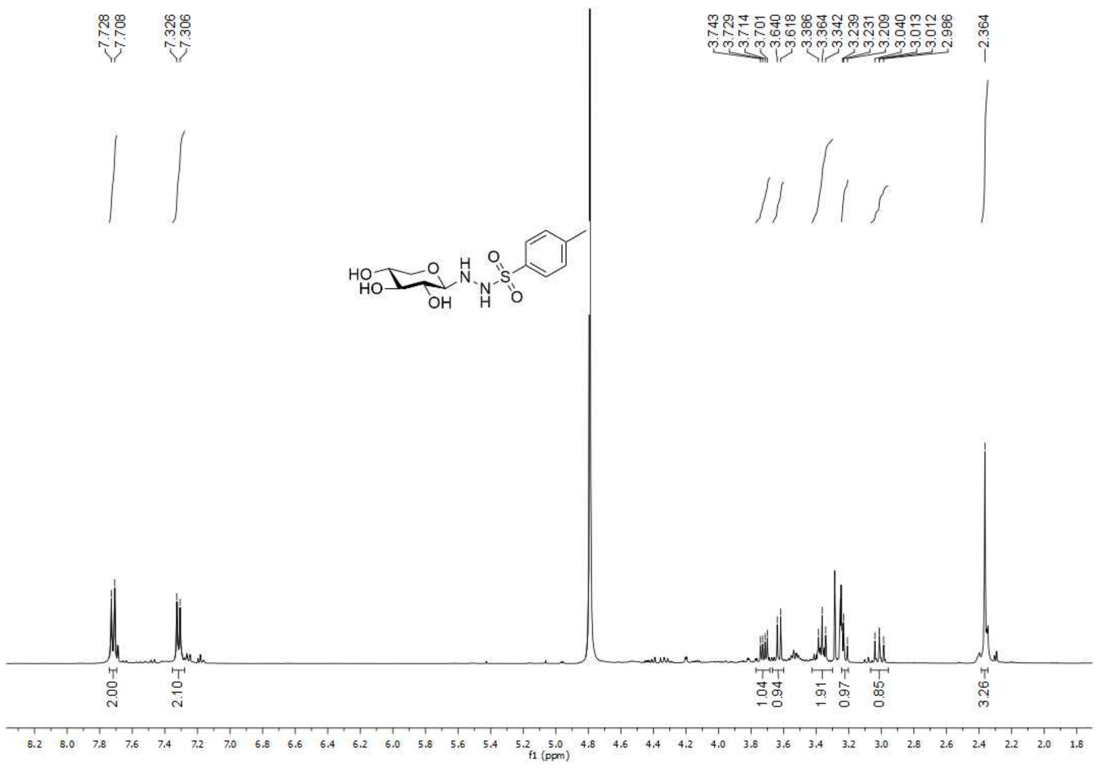

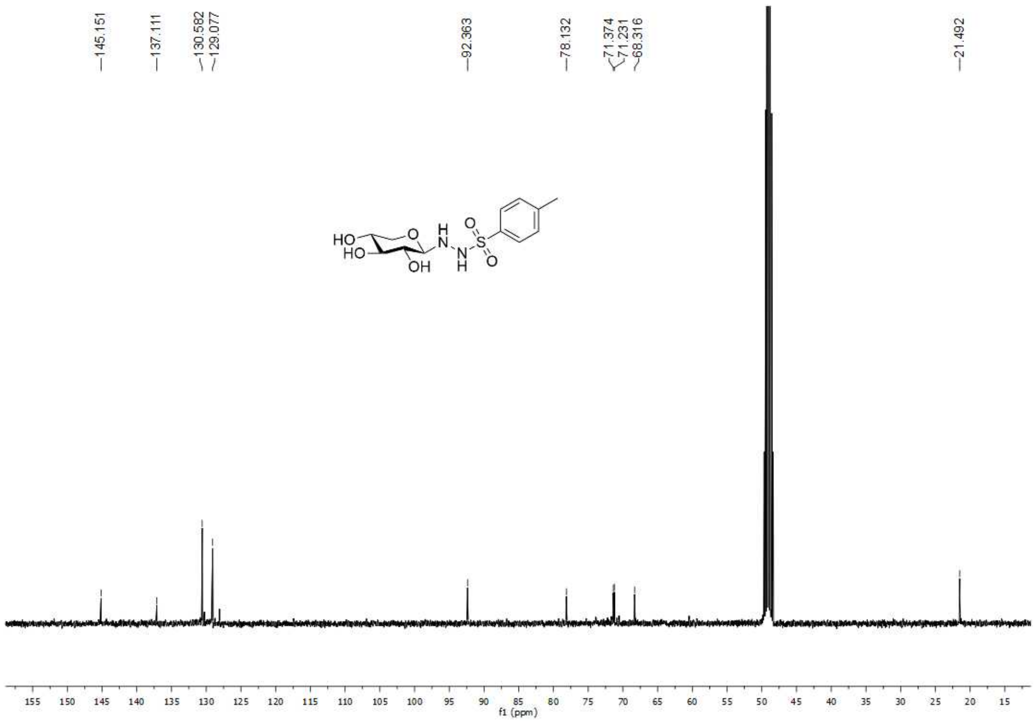

^{1}H NMR, ^{13}C NMR and^{31}P NMR of D-Xylose-1-P

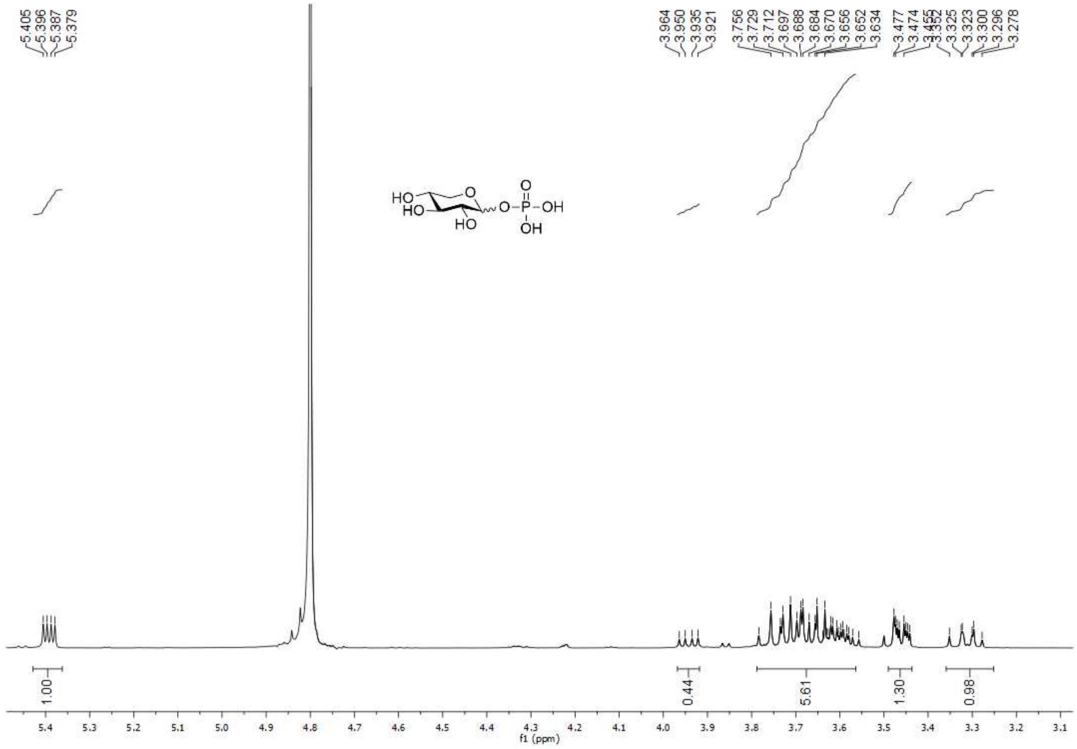

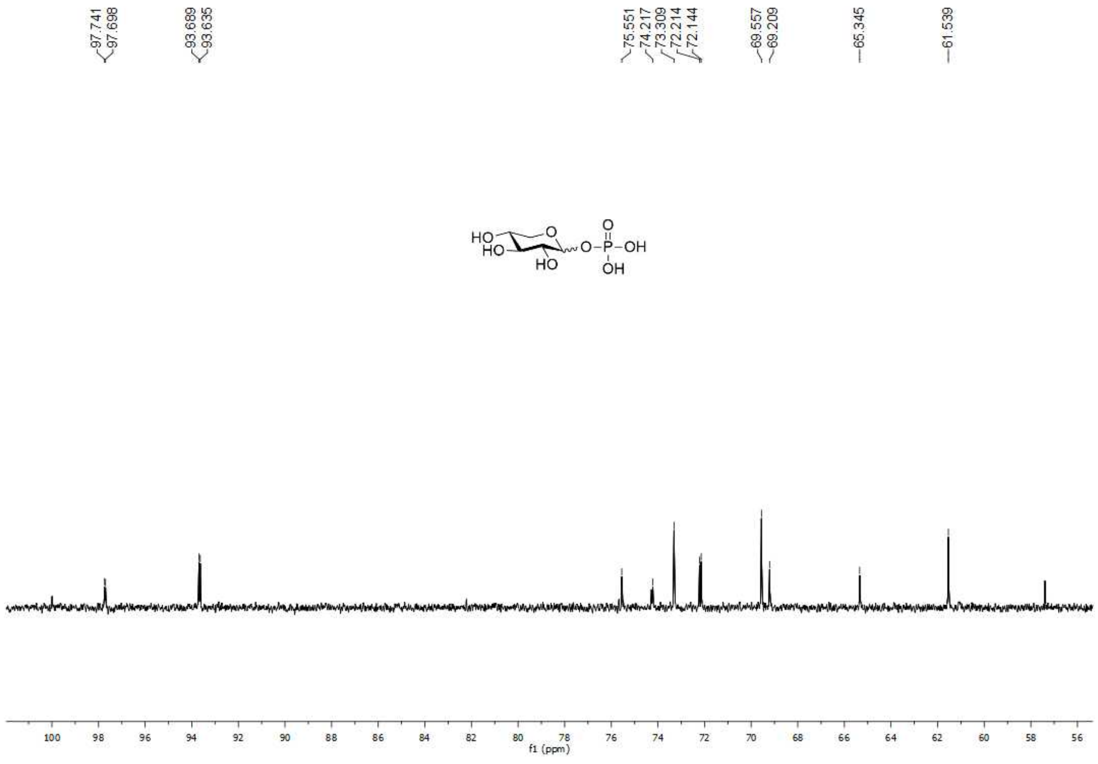
97.741
97.698
93.689
93.635
75.551
74.217
73.309
72.214
72.144
69.557
69.209
65.345
61.539
f1 (ppm)

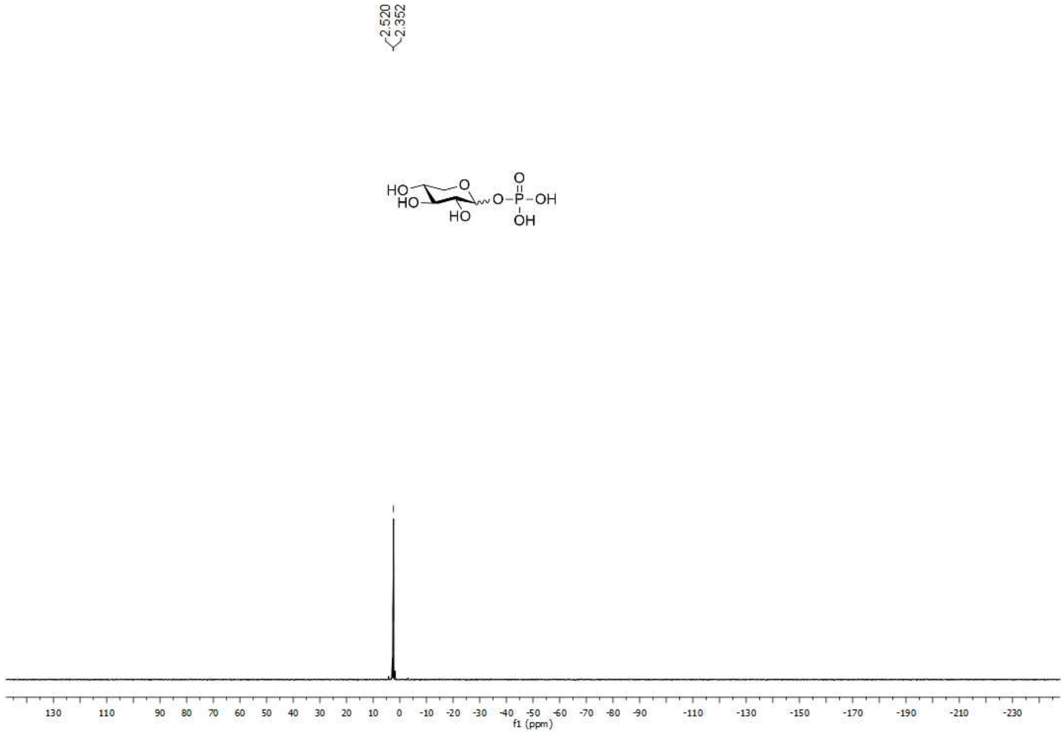
2.520
2.352
f1 (ppm)

^{1}H NMR, ^{13}C NMR and ^{31}P NMR of UDP-α-D-Xylose

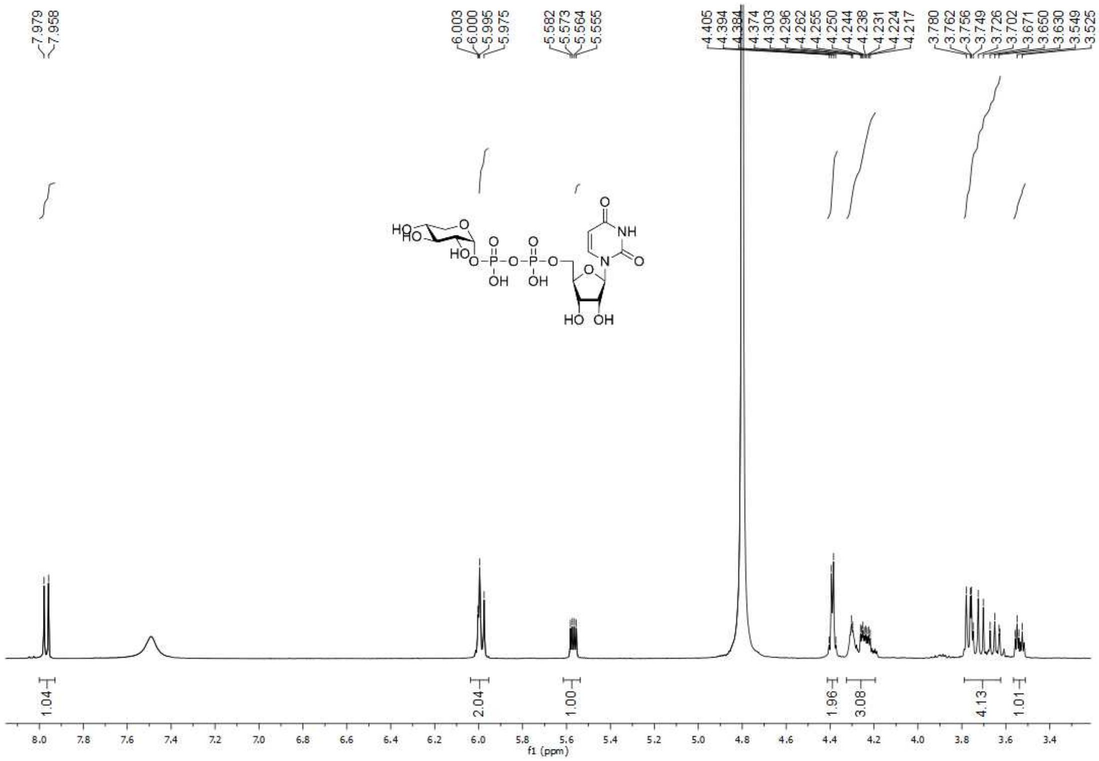

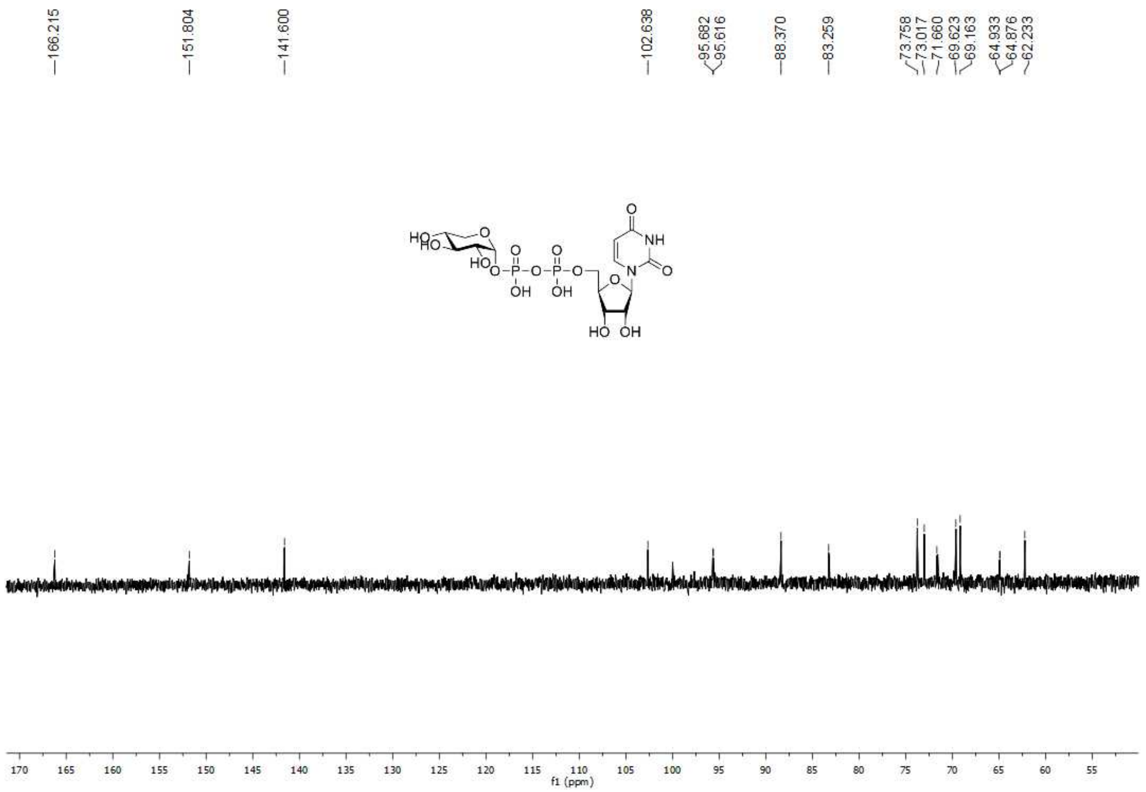

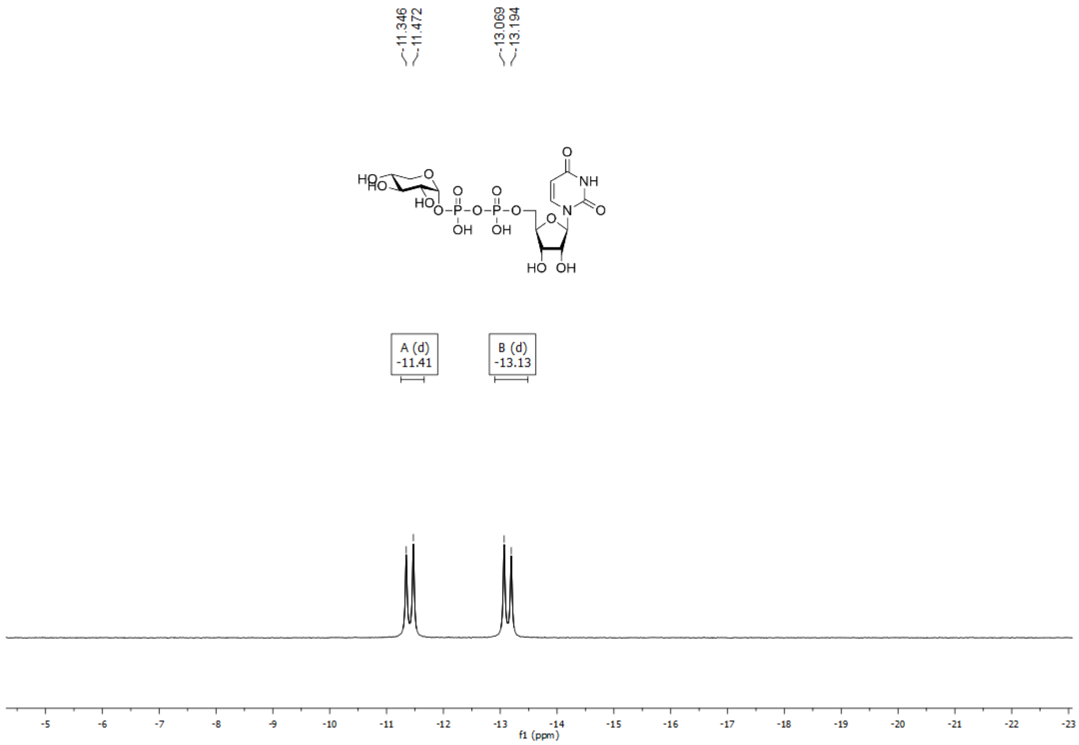

^{1}H NMR and ^{13}C NMR of TSH-L-Arabinose

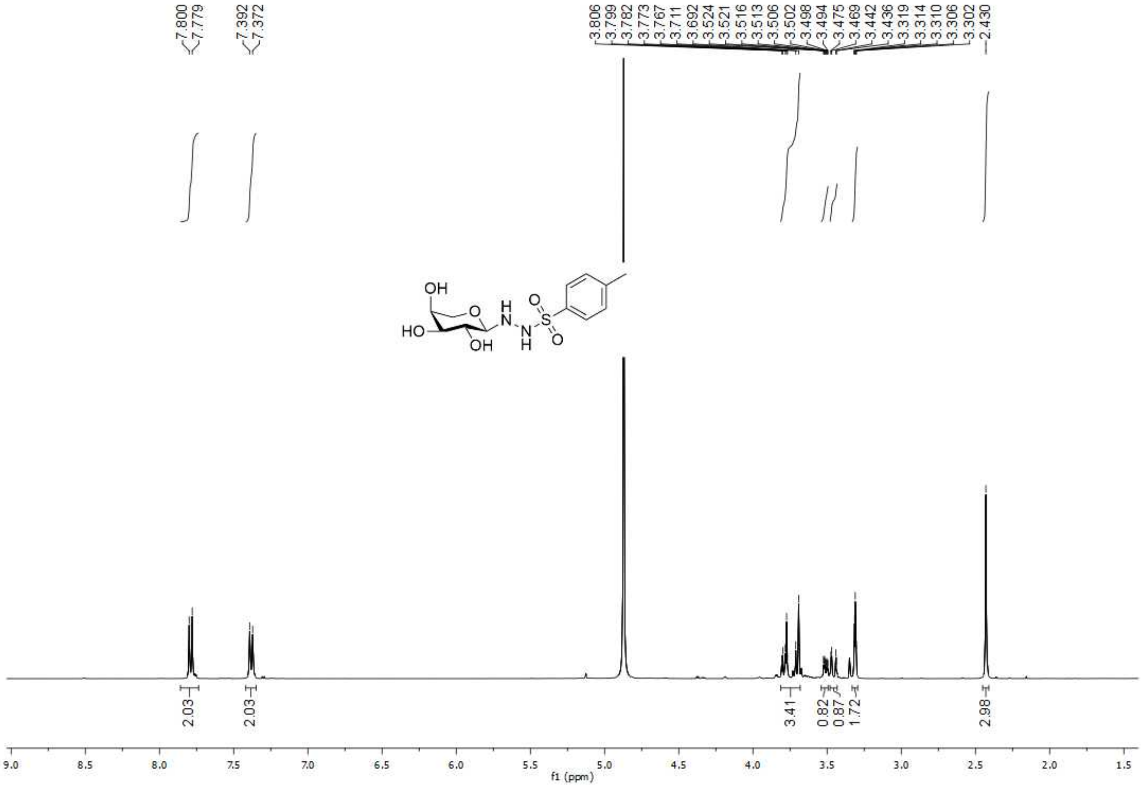

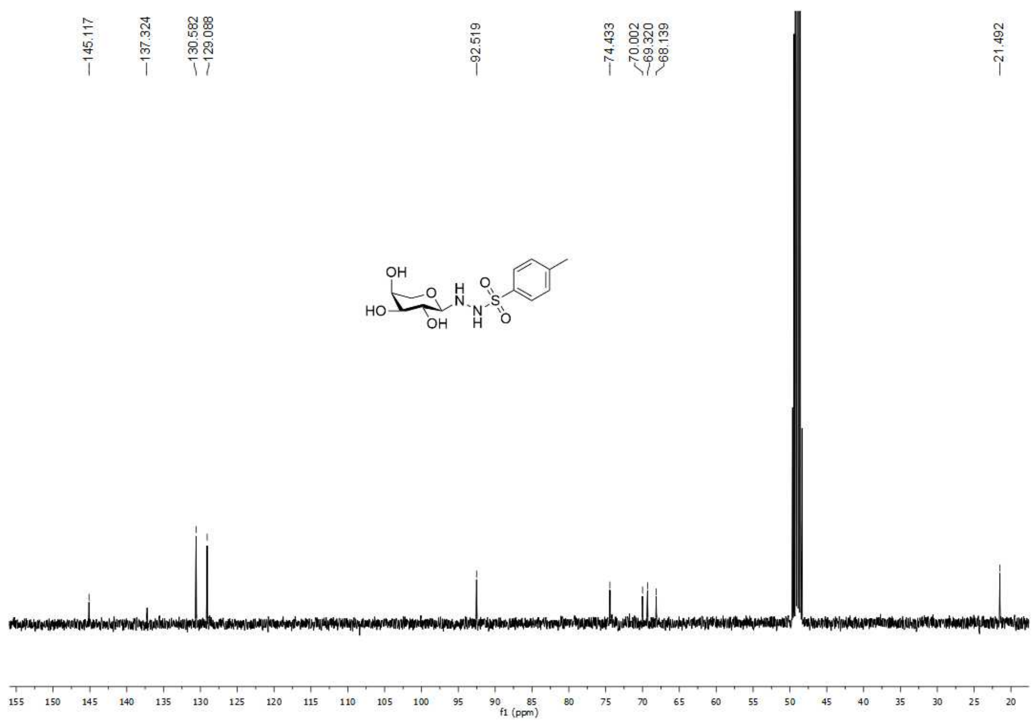

^{1}H NMR, ^{13}C NMR and ^{31}P NMR of L-Arabinose-1-P

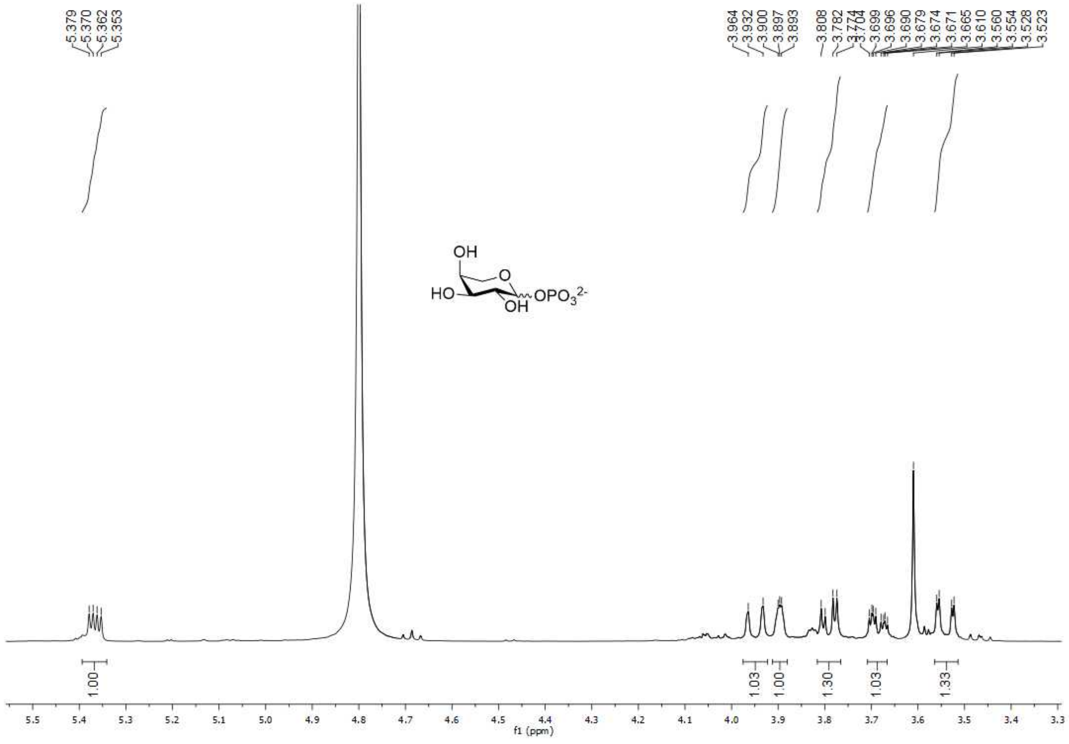

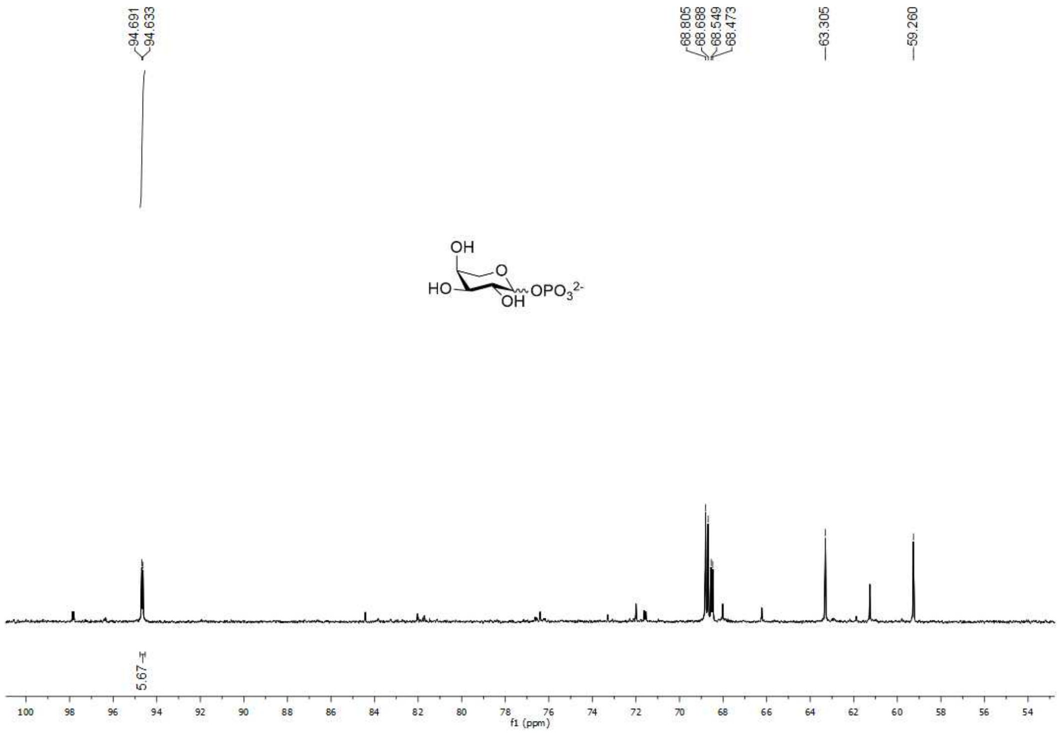
94.691
94.633
68.805
68.688
68.549
68.473
63.305
59.260
OH
HO
OH
OPO$_3^{2-}$
5.67
f1 (ppm)

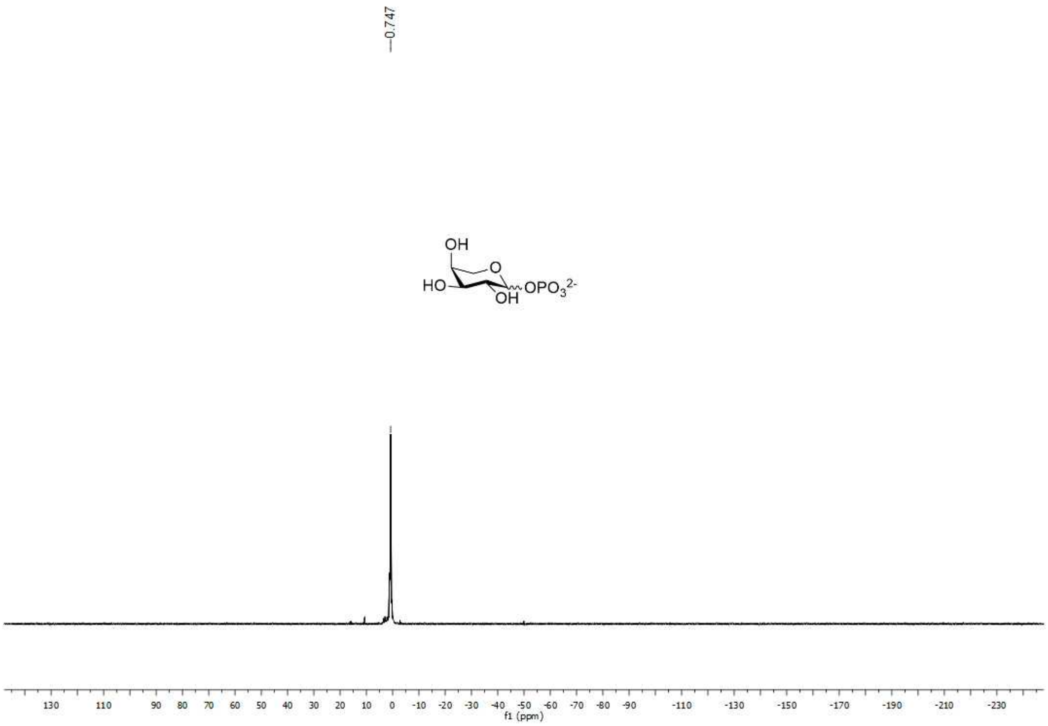
0.747
OH
HO
OH
OPO$_3^{2-}$
f1 (ppm)

^{1}H NMR, ^{13}C NMR and^{31}P NMR of UDP- β -L-Arabinose

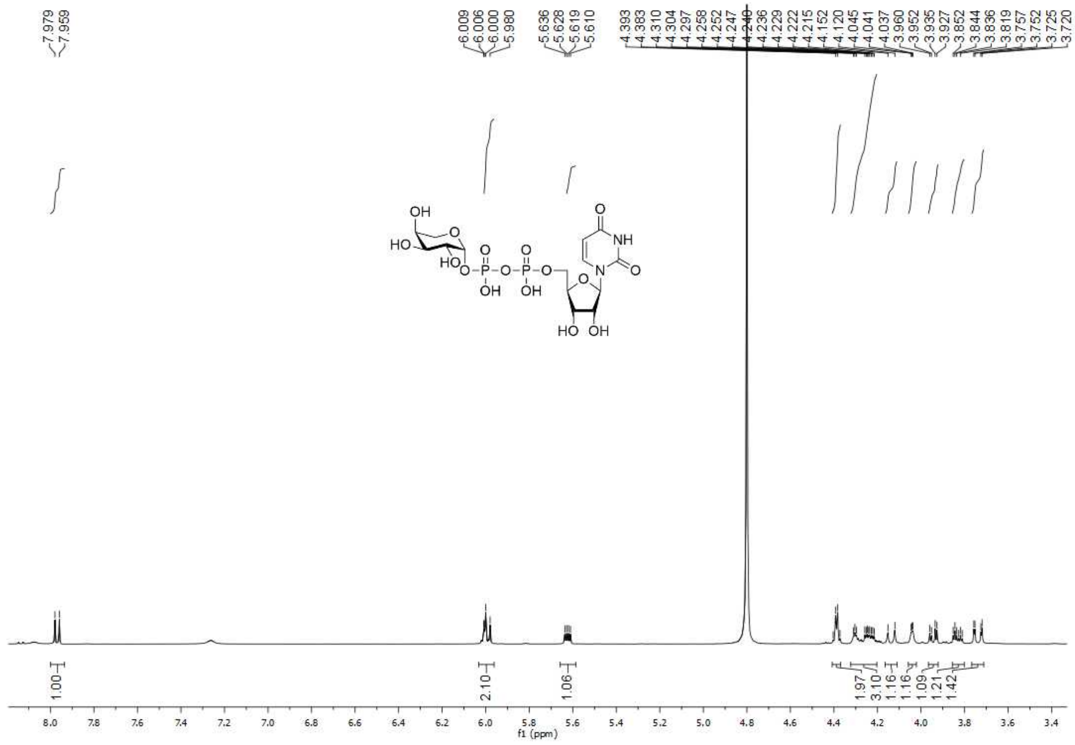

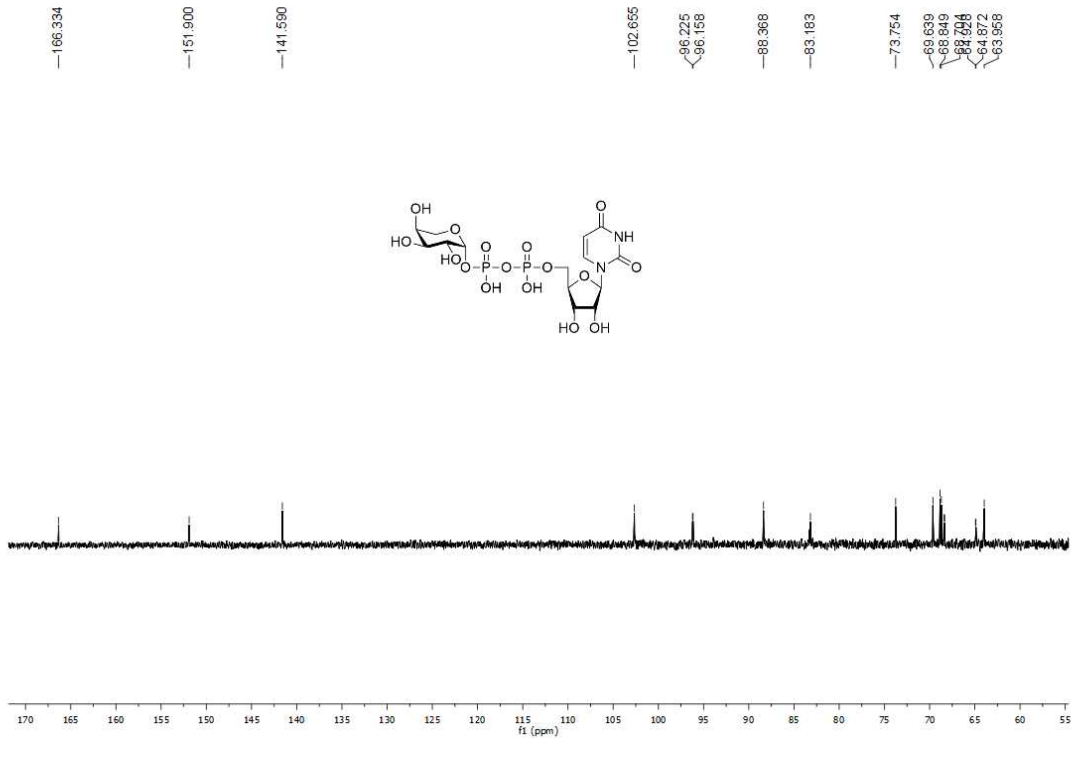

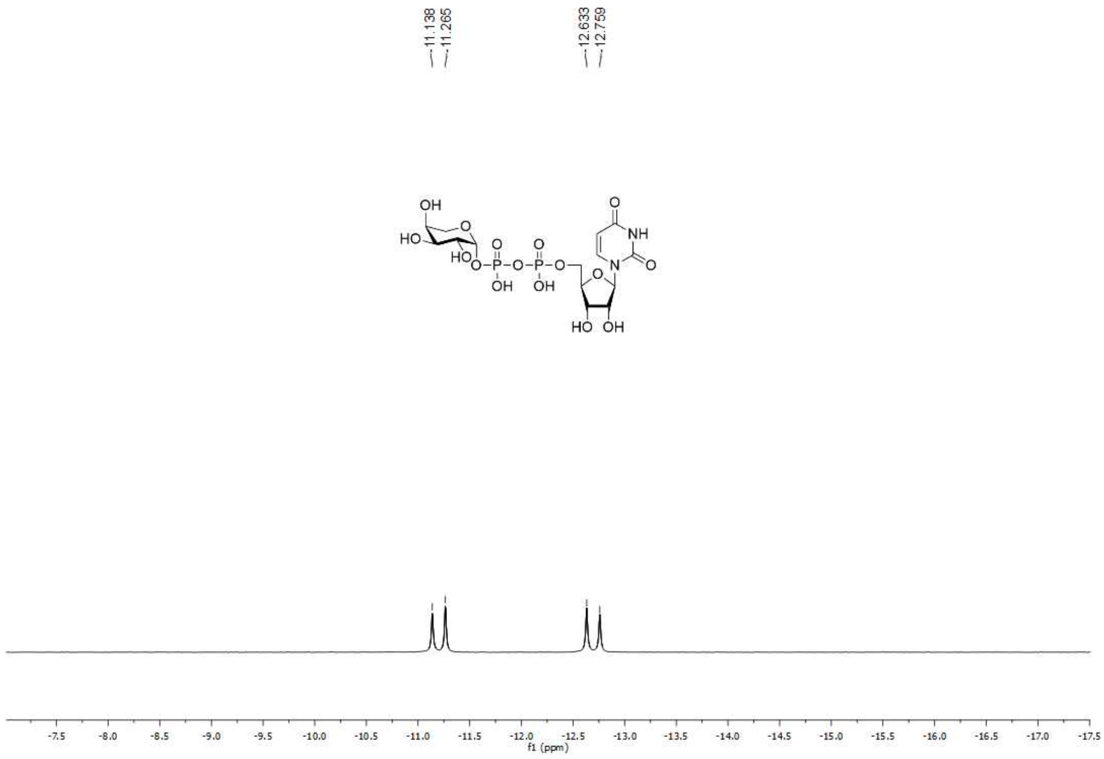

HRMS of UDP-α-D-Xylose

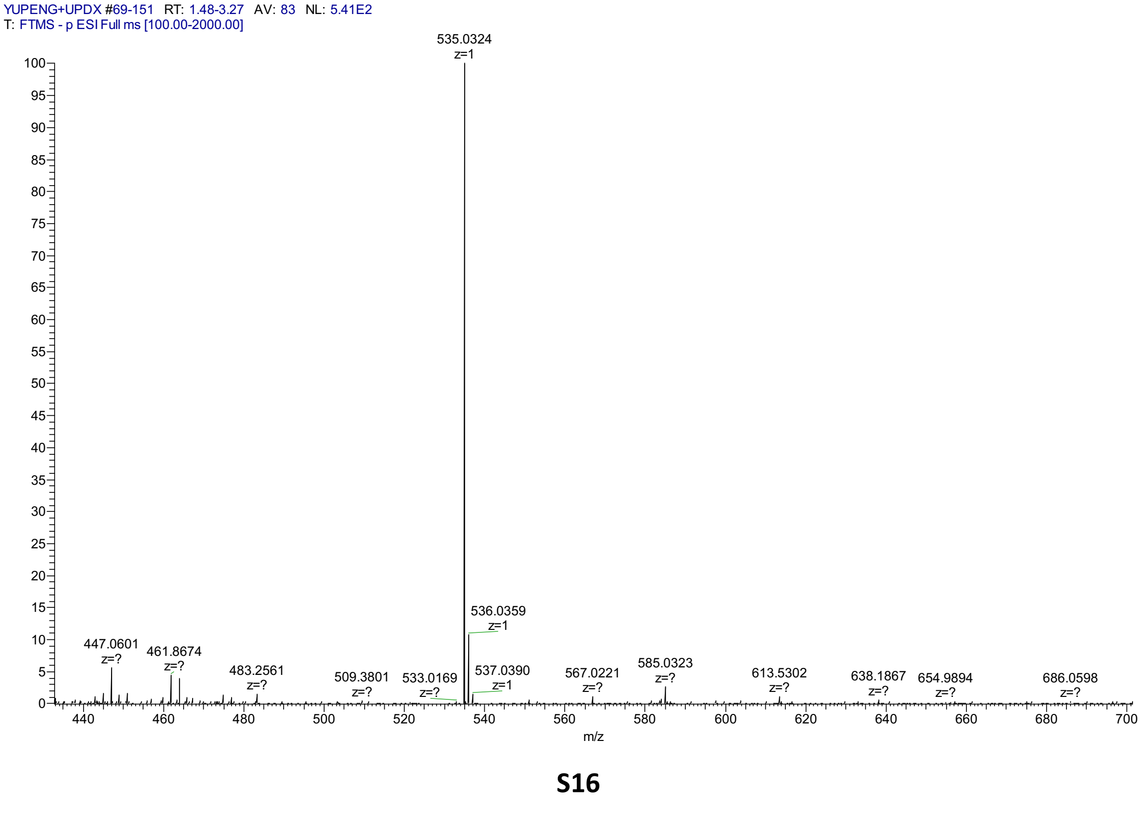

HRMS of UDP-β-L-Arabinose

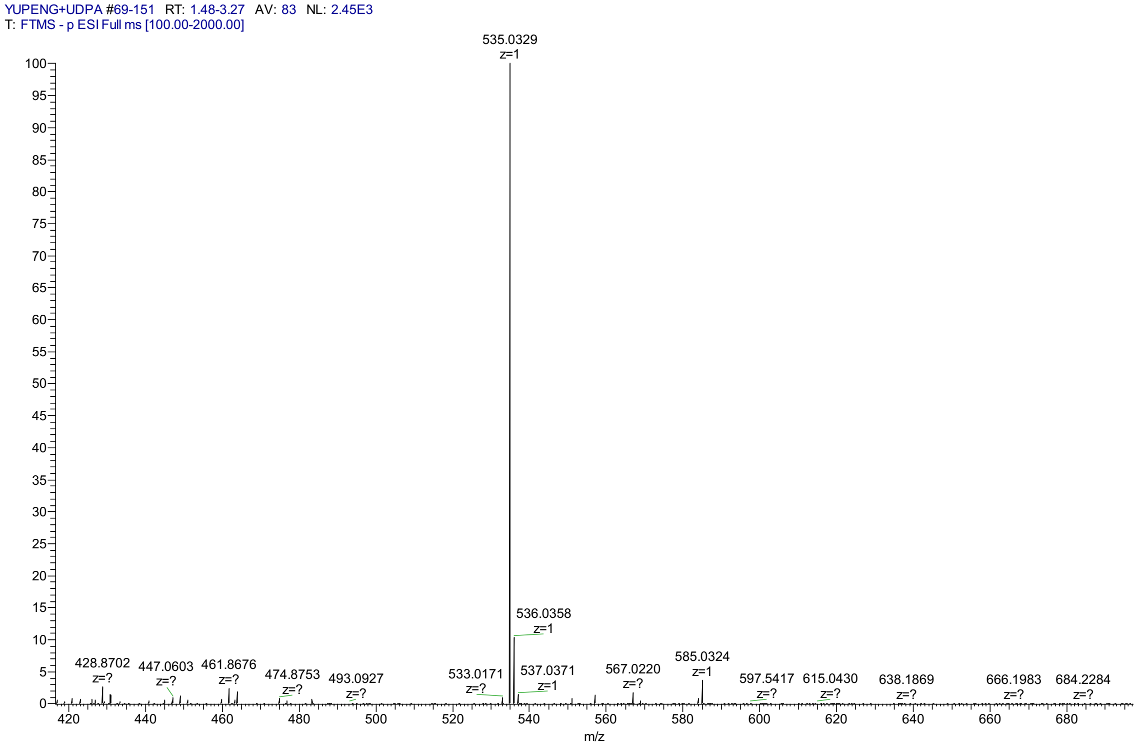

Supplement: Supplementary file 1 [file Data_Sheet_1.pdf]
